# Nonpolar Lipids Contribute to Midday Fogging During Scleral Lens Wear

**DOI:** 10.1167/iovs.64.1.7

**Published:** 2023-01-11

**Authors:** Maria K. Walker, Laura S. Bailey, Kari B. Basso, Rachel R. Redfern

**Affiliations:** 1 College of Optometry, The Ocular Surface Institute, University of Houston, Houston, Texas, United States; 2 Department of Chemistry, University of Florida, Gainesville, Florida, United States

**Keywords:** scleral lens, midday fogging, lipids, tears, fluid reservoir

## Abstract

**Purpose:**

To determine correlations between lipids in the fluid reservoir (FR) and the severity of midday fogging (MDF) in scleral lens (SL) wear.

**Methods:**

SL neophytes were recruited to wear custom SL for 4 days, examined after 8 hours on days 1 and 4. Lens vault and MDF were quantified from anterior segment optical coherence tomography (AS-OCT), and the FR was collected and analyzed by liquid chromatography–tandem mass spectrometry (LC-MS/MS). Relative abundance of lipids was compared to MDF scores using nonparametric correlation testing (Spearman rank). Ocular surface and SL fitting characteristics (lens vault, fitting curves) were likewise compared to MDF.

**Results:**

Thirteen participants (26 eyes, 69% female, 28 ± 9 years old) were included in this study. MDF severity after 8 hours of SL wear was 33 ± 29 units on day 1 and 28 ± 24 units on day 4 (*r* = .94; *P* < 0.01). Twelve samples were analyzed using LC-MS/MS, and a total of 170 distinct lipid species were detected. The lipid classes with greatest correlation to MDF were the wax esters (*r* = .73, *P* = 0.01), cholesteryl esters (*r* = .59; *P* = 0.049), and triacylglycerols (*r* = .64, *P* = 0.03). Polar lipids were observed abundantly in all samples. None of the measured ocular surface or fitting outcomes were correlated to MDF.

**Conclusions:**

Nonpolar lipids were the greatest contributors to MDF among these normal participants. Polar lipids may be due to cellular debris, although they do not appear contributory to MDF.

Scleral lenses (SLs) are large-diameter gas-permeable contact lenses used primarily to manage irregular corneal astigmatism^1^^–^^7^ and ocular surface diseases.^8^^–^^17^ They vault over the cornea, including the limbus, and land peripherally on the bulbar conjunctival tissue, trapping a thick fluid reservoir (FR) between the cornea and posterior lens surface.

The success of SLs in providing optimal vision and comfort in diseased patients is well established,^5^^,^^6^^,^^18^^–^^25^ and their expansion into a more mainstream market is ongoing.^26^^,^^27^ However, this distinctive system of contact lens wear can present with complications such as infection and tissue changes,^28^^–^^31^ as well as complications within the FR.^28^^,^^29^^,^^32^^–^^36^ The FR is formed by the mixing of the application solution (e.g., sterile saline), used to fill the concave portion of the SL, with the tears. There is an overall lack of understanding in the eyecare community with regards to the FR, which bears little resemblance to a normal tear film and is instead a deep reservoir, ranging from 100 to 1000 µm in depth over different areas of the cornea in successful SL wearers.^37^^,^^38^

The natural tear film, which is approximately 10 µm thick, contains proteins, lipids, electrolytes, gases, and metabolites in a primarily aqueous solution with concentrated nonpolar lipids superficially to reduce evaporation at the air interface. The proteins and metabolites, largely produced by the lacrimal gland,^39^ provide nutrition, antimicrobial protection, and other biological support to the ocular surface.^40^ The lipids, predominantly secreted from Meibomian glands^41^^–^^45^ but also originating from ocular surface secretory cells,^43^^,^^46^ reduce tear evaporation and also have other physiologic functions.^46^^,^^47^ Once a SL is applied to the eye and mixes with the tears to become the FR, the thickness of the tear fluid expands (from 10 to ≥100 um), inherently altering the physical properties of the fluid but also impacting the composition and concentration of the tear analytes. Once on the eye, there is variable evidence of tear exchange beneath a SL, but it is generally believed that the FR has minimal exchange with the outside tears during SL wear.^35^^,^^48^^–^^50^

A common complication of SL wear is midday fogging (MDF), in which debris accumulates in the FR during lens wear (Fig. 1). MDF is reported in an estimated 26% to 46% of SL wearers (McKinney A, et al. *IOVS* 2013;54(15):ARVO E-Abstract 5483),^28^^,^^51^^,^^52^ resulting in reduced vision and unknown physiologic effects.^28^^,^^51^^,^^52^^–^^54^ Some clinicians have hypothesized that MDF occurs due to compression of goblet cells in the conjunctiva, leading to excessive mucin secretion into the FR,^55^^,^^56^ although this has not been substantiated. An alternate hypothesis, supported by a pilot study conducted in 2014,^57^ suggested that lipids may be present in greater abundance in MDF, although no specific classes were identified. There could also be cellular contribution to the FR, such as leukocytes (i.e., neutrophils), which have been detected in the FR in patients with and without MDF.^51^ Determining the composition of the FR in MDF is an important step in understanding why it occurs and how it can be mitigated, and this study aims to quantify the lipids that contribute to MDF using mass spectrometry (MS).

**Figure 1.**
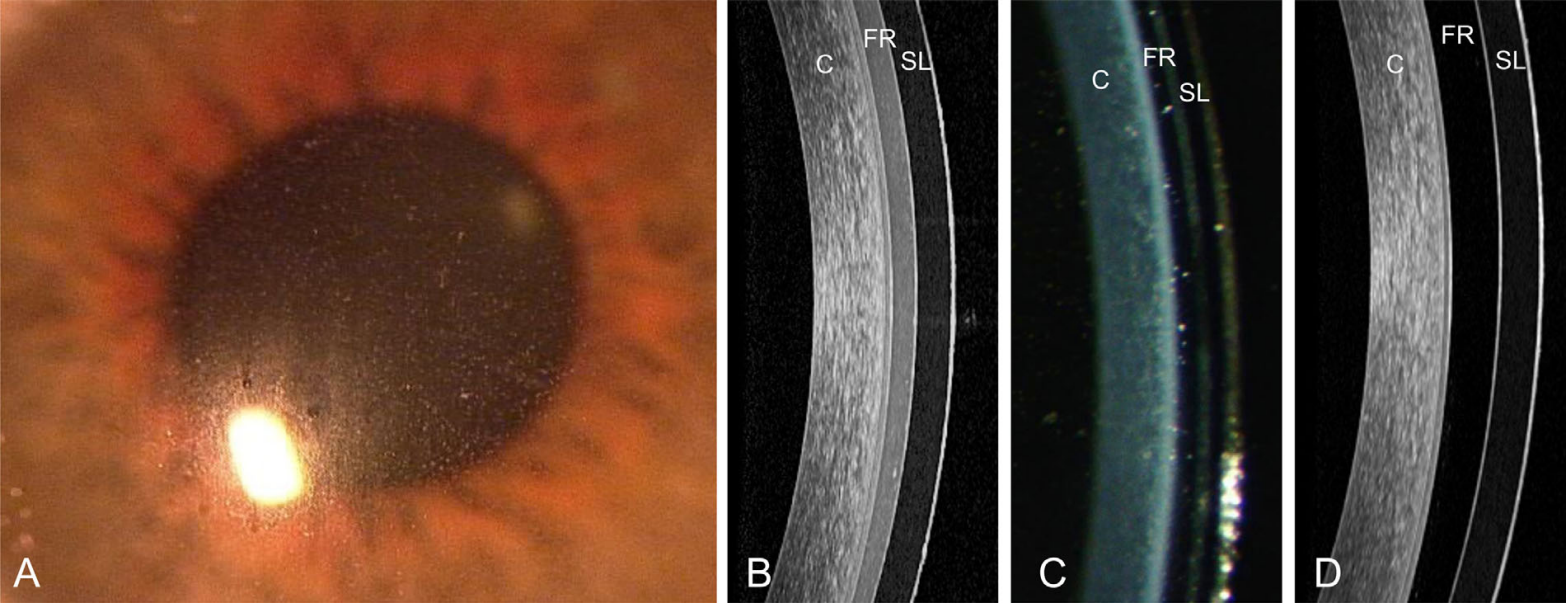
MDF in SL wear. White light biomicroscopy (10×) shows debris visible over the pupil in a patient wearing SLs who is experiencing MDF (**A**). An OCT image (**B**) and white light (40×) biomicroscope optic section (**C**) show the debris in the FR. An OCT image with no MDF is shown for comparison (**D**). C, cornea.

Liquid chromatography–tandem MS (LC-MS/MS) is a sensitive and comprehensive tool for detecting proteins, lipids, and other analytes from solution and has been used to analyze tear samples.^58^^,^^59^ Using LC-MS/MS, this pilot investigation explores the major lipids contributing to MDF, correlating them to the increasing severity of MDF and expanding the understanding of this common complication of SL wear.

## Methods

This study was compliant with the tenets of the Declaration of Helsinki and approved by the University of Houston's Institutional Review Board. All potential participants were provided an explanation of the study, and the possible side effects of participation were reviewed; informed consent was signed prior to enrollment.

### Participants

A total of 13 normal participants were recruited and study visits were performed at the University of Houston, College of Optometry (UHCO). Inclusion criteria were normal, soft contact lens–wearing adults between ages 20 and 50 years, with normal ocular surface health, including the cornea and conjunctiva. Exclusion criteria were a history of ocular surface disease or surgery, including refractive surgery, within the past 2 years; greater than trace level of corneal staining in either eye; or self-reported ocular allergies, corneal erosions, or ocular infection within the past year. Any participants who reported use of ocular medication, including artificial tears, within the previous week were excluded. Participants were also excluded if they reported an inability to apply and wear SL for 8 hours per day or a sensitivity to Fluress (Akorn Inc., Gurnee, IL) ophthalmic drops. Soft contact lens wear was discontinued at least 3 days prior to beginning the experiments.

### Scleral Lens Fitting and Evaluation

Scleral topography (sMap; Precision Ocular Metrology, NM, USA) was completed, and custom SLs (Europa; Visionary Optics, Front Royal, VA, USA) were ordered based on topography and refractive calculations. All SLs were manufactured in high Dk (125 barrers) gas-permeable plastic (Optimum Extreme, Contamac, UK), and toric or spherical landing curvatures were designed and adjusted as needed in the preexperimental fitting and training sessions. The aim of all SL fits was corneal clearance with a 100- to 300-µm central vault and minimal conjunctival compression.

The day prior to beginning SL wear, participants reported to UHCO, where the SLs were applied, and anterior segment–optical coherence tomography (AS-OCT) (Visante OCT; Carl Zeiss, Oberkochen, Germany) was performed to determine baseline corneal vault. Lens vault was calculated using software calipers, measured as the distance between the posterior SL surface and the anterior apical corneal surface in µm. Visual acuity (VA) was measured as a safety outcome, using a Snellen acuity chart in a high-contrast, high-luminance setting. After baseline measurements, the SLs were removed, dispensed to the participant, and disinfected overnight using a hydrogen peroxide disinfection system (Clear Care; Alcon Laboratories, Fort Worth, TX, USA). This system is a one-step (i.e., does not require rubbing) system in which the SLs are placed into the lens case for 6 or more hours, during which time disinfection occurs as the peroxide is neutralized; this method was chosen to maximize compliance with the disinfection protocol. Careful application training was done at the dispense visit to ensure that the participants were able to apply the SLs without catching them on the lids or inducing a bubble. The next morning, participants applied the SLs 8 hours prior to the scheduled experimental visit using sterile saline, buffered with boric acid and sodium borate (Purilens; LifeStyle Co., Freehold, NJ, USA), as the application solution. Participants reported to UHCO 8 hours after applying the SLs for the experimental visit, where VA was measured (Snellen) and the SLs were evaluated in white light biomicroscopy to grade global and limbal hyperemia (Cornea and Contact Lens Research Unit [CCLRU] scale).^60^ AS-OCT images were also acquired prior to SL removal.

### MDF Quantification

The AS-OCT images used to quantify MDF were taken as single-line raster scans near the geometric center of the cornea, which included the cornea, FR, and SLs in the image. To quantify MDF severity, AS-OCT images were processed using a custom ImageJ (National Institutes of Health, Bethesda, MD, USA) protocol to calculate the net gray value in the FR in calibrated optical units (Supplementary Fig. S1). MDF scores were determined at each experimental visit, and the final MDF score for each eye was averaged from days 1 and 4. The image analysis was completed by two masked examiners for each image, and the repeatability of the scoring method was determined as the within-subject standard deviation (SD).^61^^,^^62^ In addition, MDF was graded subjectively from 1 to 5 and then designated MDF (score of 4 or 5) or non-MDF (score of 1, 2, or 3) for group comparisons (Supplementary Figs. S2 and S3).

### Fluid Reservoir Collection

After biomicroscopy and AS-OCT imaging, the FR was collected by a single investigator (MW) using a method described previously.^32^ In this method, the SL is carefully removed while the participant's chin is tilted downward with the eye parallel to the ground, and then the FR is removed from the concave portion of the lens using a micropipette. Samples were transferred to DNA LoBind microcentrifuge tubes and frozen at –80°C for up to 6 months until analysis. After SL removal, corneal and conjunctival staining was graded using biomicroscopy (CCLRU scale). After the visit on day 1, participants were redispensed the SLs and instructed to wear them 8 hours per day for the next 3 consecutive days, disinfecting overnight using the hydrogen peroxide system and applying with saline each morning, returning for a repeat visit on day 4. A total of 12 pooled tear samples with ranging MDF severity were sent to the Mass Spectrometry Research and Education Center (MSREC) at the University of Florida Department of Chemistry for lipid analysis.

### Lipid Identification and Analysis

From each available FR sample, 20 µL was shipped on dry ice to the MSREC and a total of 2 µL sample and 5 µL SPLASH internal standard (Avanti Polar Lipids, Alabaster, AL) were extracted twice using 5 volumes of ice-cold isopropanolol. The solution was thoroughly mixed and centrifuged (Minifuge, United Laboratory Plastics, St. Louis MO) for 5 minutes. The combined supernatant was dried under vacuum to completeness and reconstituted in 100 µL methanol with 1% chloroform. Samples were analyzed by reversed-phase chromatography (PepMap C18; 300 µm × 15 cm, 2 µm, 100 Å; ThermoScientific, Waltham, MA) using a 90-minute method using the mobile phases (A) 60/40 acetonitrile/water and (B) 90/8/2 isopropanol/acetonitrile/water, both containing 10 mM ammonium formate and 0.1% formic acid. Samples were washed on a trap of the same material (ThermoScientific; 0.5 × 5 mm, 5 µm) at 98% acidified acetonitrile for 5 minutes while the column was equilibrated at 50% B. The preconcentrated sample was then separated using the following method: 5 to 50 minutes, 50% to 75% B; 50 to 70 minutes, 75% to 98% B; and 70 to 90 minutes, 98% B. Total lipid concentration was determined by a sulfo-phospho-vanillin assay,^63^ and the sample loading was normalized for 0.1 µg total lipid content. Detection was performed on a Bruker (Billerica, MA) QTOF-MS (Impact II quadrupole-quadrupole-time-of-flight mass spectrometer), with data-dependent MS/MS programmed for collision-induced dissociation of singly and doubly charged ions between *m/z* 500 and 1500 using a mass window of *m/z* 2 and mass-dependent collision energies from 25 to 40 eV. Active exclusion was programmed for exclusion after a single spectrum with release after 3 minutes unless the ion was twice as abundant. A preceding sodium formate infusion ensured mass accuracy throughout the sequence, and a pooled quality control sample ensured instrument reproducibility and sensitivity over the acquisition.

The resultant lipid data were then processed in Metaboscape (Bruker, v.5.0). Spectra were searched using LipidBlast (v.66),^64^ MSDIAL,^65^ and SimLipid (Premier Biosoft International, San Francisco, CA; v.6.05).^66^ Ions without an MS/MS spectra were annotated using Lipid Maps (2019).^67^ Wax ester assignments were performed manually.^68^ Postprocessing sorting and analysis were done in Microsoft Excel (Microsoft, Redmond, WA, USA) and GraphPad Prism (GraphPad Software, La Jolla, CA, USA).

### Statistical Analysis

The Shapiro–Wilk test for normality was used for all outcomes, and significance was set at *P* ≤ 0.05. MDF scores were calculated for each image using ImageJ and are reported as calibrated units of optical density (units). Relative abundance of lipids was calculated and compared to MDF scores using the Spearman rank to test correlation of nonparametric data. To evaluate the correlation between MDF and the other outcomes (lens vault, lens settling, VA), right eyes only were selected for the analysis, and the Spearman rank test was used.

## Results

### Participant Demographics and Scleral Lens Parameters

A total of 13 participants (26 eyes) completed this pilot study (Table 1). The SLs were designed to vault the cornea, including limbus, and land without impingement or blanching of blood vessels on the conjunctival surface. The SL diameter for 70% of participants (*n* = 9) was 16.0 mm, and those participants with smaller or larger than approximately 11.8-mm iris diameter were made SLs with smaller (15.5 mm, *n* = 1) or larger (16.2 mm, *n* = 1; 16.5 mm, *n* = 2) diameters. All lenses were manufactured with a central 8.5-mm-wide optic zone and a 2.1-mm-wide adjacent peripheral curve, with variable central and peripheral radii of curvature depending on the power and sagittal depth (SAG) of the lens. Mean ± SD SAG was 4368 ± 244 µm, and 18 lenses were designed with toric (rotationally asymmetric) landing zones with a mean ± SD toricity of 127 ± 114 µm. Mean ± SD VA at lens dispense was −0.03 ± 0.13 (20/18 Snellen acuity) and was slightly reduced to −0.01 ± 0.05 (about 20/20 Snellen) after 8 hours of SL wear on day 1 and –0.01 ± 0.10 on day 4. There was no correlation between change in VA and increasing MDF scores (*r* = .24; *P* = 0.23).

**Table 1.** Participant Demographics and Scleral Lens Parameters

Participant Demographic	Outcome
Age, mean ± SD, y	28 ± 9
Gender, female, *n* (%)	9 (69)
Hours of lens wear: day 1, mean ± SD	8.7 ± 1.5
Hours of lens wear: day 4, mean ± SD	8.9 ± 1.4
VA at dispense (calculated logMAR), mean ± SD	−0.03 ± 0.13
VA after 8 hours of lens wear: day 1, mean ± SD	−0.01 ± 0.05
VA after 8 hours of lens wear: day 4, mean ± SD	−0.01 ± 0.10
SL parameters	
Brand	Europa
Manufacturer	Visionary Optics
Material	Optimum Extreme (Contamac)
Dk, barrer	125
Power range, diopter (spherical equivalent)	+1.00 to −7.50
Diameter range, mm	15.5–16.5
SAG range, µm	3980–4815
Toric landing zone, lenses, *n* (%)	18 (69)

### AS-OCT Analysis: Vault and MDF Scoring

AS-OCT images were used to determine SL vault and MDF for all 26 eyes, although 1 eye settled 240 µm and had no clearance after 8 hours of lens wear so was not analyzed for MDF. Lens vault at SL dispense (0 hours wear) was 293 ± 84 µm, and the lenses settled an average of 81 ± 75 µm on day 1 and 88 ± 42 µm on day 4. The MDF severity graded using ImageJ was 33 ± 29 units (range, 7–96) on day 1 and 28 ± 24 units (range, 6–91) on day 4. One participant had one eye that was considered in the non-MDF group (score, 26) and the fellow eye was in the MDF group (score, 39). All other participants either had MDF in both eyes or did not, and day 1 and 4 MDF scores were strongly correlated both within and between eyes (*r* = .94; *P* < 0.001) (Supplementary Fig. S4). Subjective MDF grading designated 7 eyes (4 participants, 27%) showing clinically significant MDF and 18 eyes (9 participants) that did not. The subjective measurements agreed with the objective scoring (see Supplementary Fig. S3), and repeatability of the objective (ImageJ) method was 0.97.

There were no significant correlations between MDF severity and SL vault, settling, or any fitting characteristics or ocular surface outcomes (Table 2). Limbal clearance, although not measured objectively, was subjectively evaluated as excessive, adequate, or minimal and was graded excessive in 4 out of 7 eyes with MDF (57%) and only 2 out of 18 eyes (11%) without MDF. Three eyes experienced blood vessel blanching in any quadrant, two eyes with MDF and one eye without.

**Table 2.** SL Fitting and Ocular Surface Health Outcomes and Their Correlations to MDF

Outcome	At SL Dispense	Post-SL Wear	Correlation to MDF^*^ *r* (*P* Value)
Landing zone toricity^†^ (µm)	114 ± 113		+0.32 (0.12)
Visual acuity (logMAR)	−0.03 ± 0.13	−0.03 ± 0.10	+0.31 (0.29)
Corneal staining	0.1 ± 0.1	0.4 ± 0.4	+0.07 (0.83)
Limbal hyperemia	0.5 ± 0.2	0.9 ± 0.9	−0.06 (0.83)
Global hyperemia	1.0 ± 0.7	1.5 ± 0.7	+0.11 (0.71)
Lens vault (µm)	285 ± 89	203 ± 77	−0.06 (0.85)
Lens vault settling (µm)	NA	86 ± 52	−0.16 (0.63)

Unless otherwise noted, data are represented as the mean ± SD.

* Correlation with MDF using Spearman rank correlation testing, right eyes only (*n* = 13).

† Mean toricity in the landing zone overlying the conjunctiva.

### Lipids

**Figure 2.**
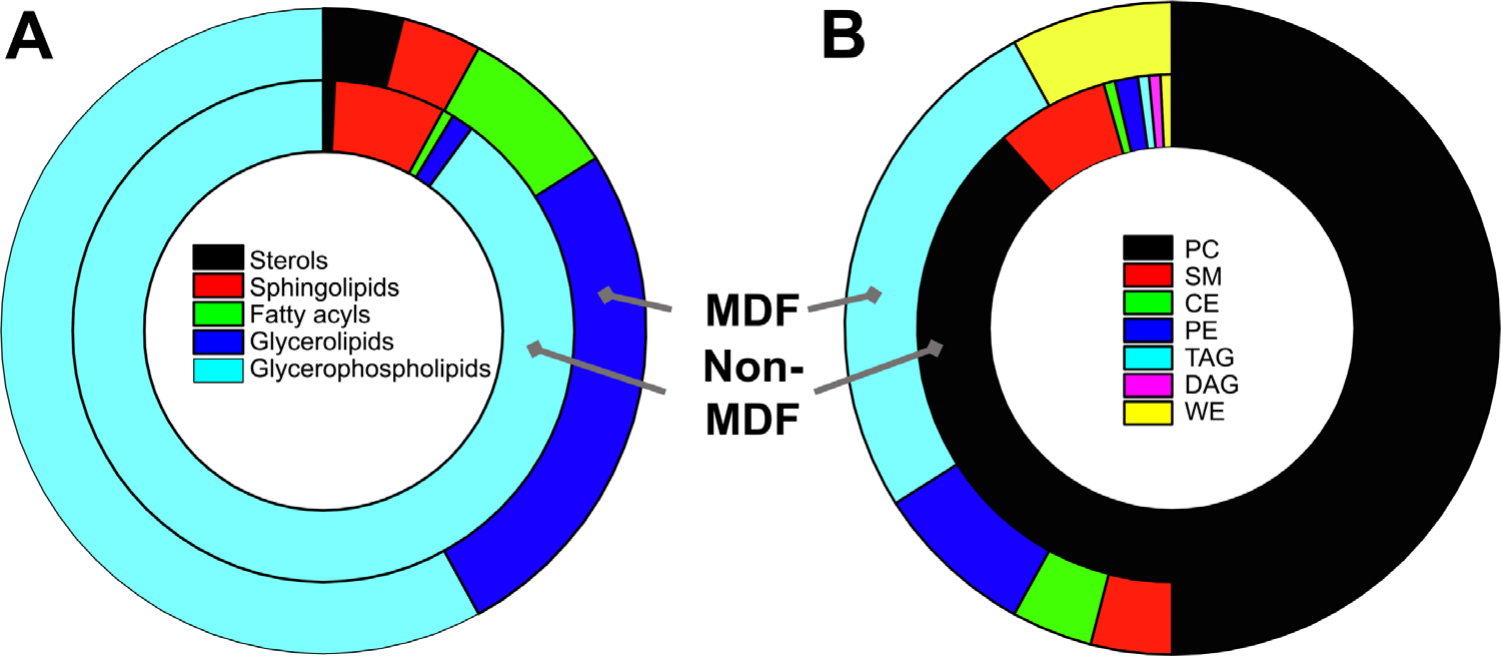
Major lipids categories and classes found in the FR. The proportion of major lipid categories (**A**) and the primary classes within them (**B**) are shown for the MDF (*outer ring*) and non-MDF (*inner ring*) samples of FR. DAG, diacylglycerol.

A total of 12 FR samples were analyzed for lipids using LC-MS/MS, with MDF scores ranging from 6 to 92 units (7 MDF, 5 non-MDF). The mean lipid concentration of the samples was 32.3 ± 32.0 µg/mL (31.0 ± 9.3 in the MDF group; 33.0 ± 13.9 in the non-MDF group) and was not correlated to increasing MDF severity (*P* = 0.83). A total of 170 distinct lipids were detected, primarily from the sterol, fatty acyl, glycerolipid, sphingolipid, and glycerophospholipid categories (see Supplementary Table S1 for raw lipid data). The fatty acyls, glycerolipids, and sterols were proportionately found more in the MDF group, while the sphingolipids and glycerophospholipids comprised most of the lipids detected in the non-MDF samples (Fig. 2A). When investigated by class (Fig. 2B), wax esters (WEs), triacylglycerols (TAGs), and cholesteryl esters (CEs) were more proportionately abundant in MDF samples, compared to the non-MDF samples, which had a larger proportion of phospholipids such as phosphatidylcholine (PC), phosphatidylethanolamine (PE), and sphingomyelin (SM).

**Figure 3.**
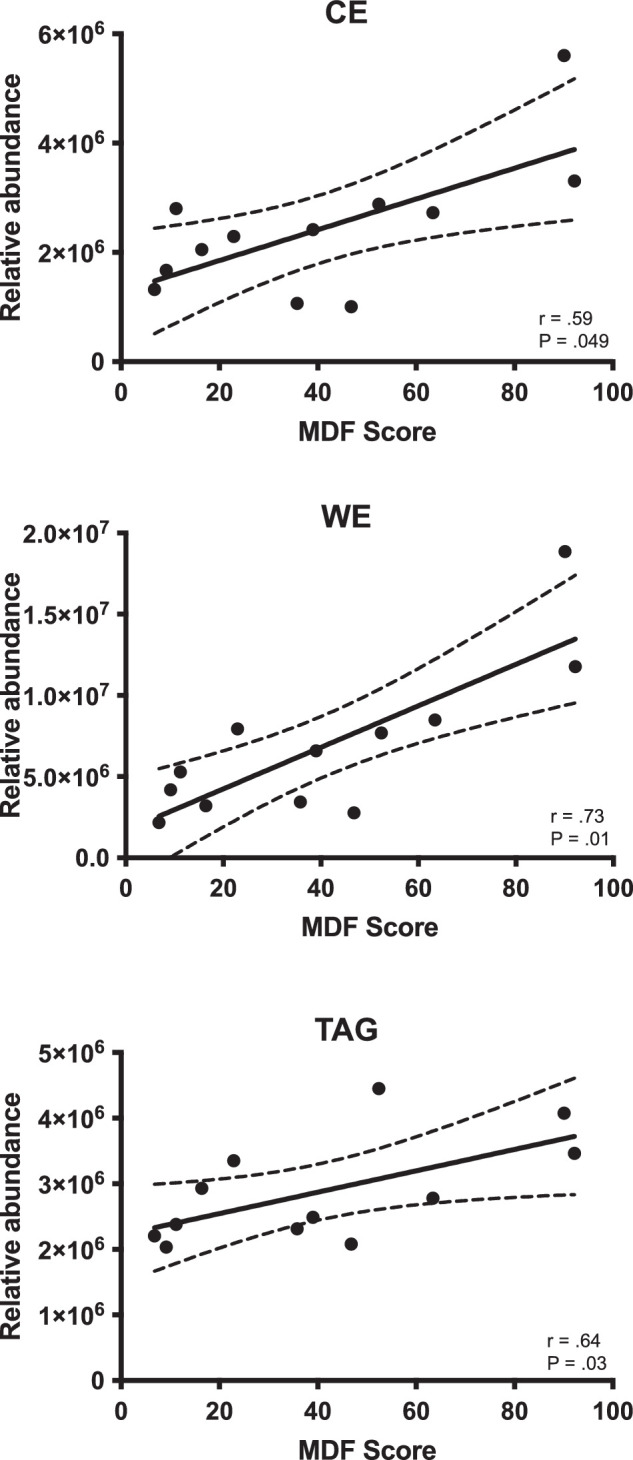
Nonpolar lipid abundances versus MDF severity. Correlation between MDF score and relative abundance of CEs, WEs, and TAGs. All three classes showed significant positive correlations to increasing MDF.

The glycerolipids comprised 26% of the lipids in the MDF samples and 2% in non-MDF. The two classes detected were the diacylglycerols, which made up <1% of all lipids, and the TAGs, which were among the most abundant lipid species detected. TAGs were positively correlated to MDF severity (*r* = .64, *P* = 0.03) (Fig. 3), and those found most abundantly in MDF were often unsaturated with long carbon chains (>55 carbons) (Table 3).

**Table 3.** Lipid Species With Greatest Correlations to MDF

Lipid Class/Species	Properties and Function	Correlation to MDF, *r* (*P* Value)
Sterols		
CE 19:0	Esters of a fatty acid with cholesterol^44^	.66 (.02)
CE 20:0; 20:1; 20:2	Hydrophobic in nature^69^	.67 (.02); .71 (.01); .71 (.01)
CE 21:0	Role in reducing tear film evaporation^44^^,^^46^^,^^70^	.73 (<.01)
CE 22:0; 22:1; 22:2	Tend to form separate phase in aqueous solution^44^	.68 (<.01); .75 (<.01); .63 (.03)
CE 23:0		.65 (.03)
CE 24:1; CE 24:2		.76 (<.01); .78 (<.01)
CE 26:2		.69 (.02)
Fatty acyls		
WE 34:2	Esters of a fatty acid and fatty alcohol^71^	.82 (.001)
WE 39:1; WE 39:2	Reduce evaporative loss^71^	.69 (.01); .74 (<.01)
WE 40:3; 40:0; 40:2	Extremely hydrophobic and high melting^70^	.78 (<.01); .65 (.03); .74 (<.01)
WE 41:0	Possible role in aiding spread of oily layer^72^	.64 (.03)
WE 42:0; 42:2	Interact with CE to form hydrophobicity^70^^,^^73^	.66 (.03); .80 (<.01)
WE 43:1; WE 43:2; WE 43:3		.71 (.01)
WE 44:1; WE 44:2; WE 44:4		.66 (.02); .76 (<.01); .78 (<.01)
WE 46:2; WE 46:3		.76 (<.01); .71 (.01)
WE 47:1; WE 47:2		.71 (.01); .75 (<.01)
WE 48:1; WE 48:2; WE 48:3; WE 49:2		.73 (<.01); .78 (<.01)
WE 50:2; WE 50:3		.77 (<.01); .76 (<.01)
Hexacosanyl palmitoleate		.76 (<.01)
Glycerolipids		
TAG 54:2	Esters of a glycerol and three fatty acids^68^	.71 (.01)
TAG 54:3	Possible role in interfacing between nonpolar lipids and aqueous tears^74^	.73 (<.01)
TAG 56:3		.92 (<.001)
TAG 56:5		.71 (.01)

Fatty acyl lipids were approximately 8% of the lipids in the MDF group and <1% of the lipids in the non-MDF group (Fig. 2). The most abundant class of fatty acyls included the WEs, which were positively correlated to MDF overall (*r* = .73, *P* = 0.01) (Fig. 3) and showed strong correlations between the unsaturated WE species (with >40 carbons) and MDF (Table 3). The ratio of saturated to unsaturated WEs ranged from .07 to .11 and was negatively correlated to MDF (*r* = −.76, *P* < 0.01), indicating greater unsaturated (relative to saturated) WEs in the MDF samples.

Sterol lipids comprised approximately 4% of total lipids in the MDF group and <1% of the lipids in the non-MDF samples (Fig. 2), primarily made up of lipids from the CE class. The relative abundance of CE was positively correlated to increasing MDF (*r* = .59; *P* = 0.049) (Fig. 3), and several individual CE species, both saturated and unsaturated with 19 to 26 carbons, showed strong correlations to MDF (Table 3). The ratio of saturated to unsaturated CE ranged from .21 to .72 and was not correlated to MDF (*r* = .21, *P* = 0.51).

**Figure 4.**
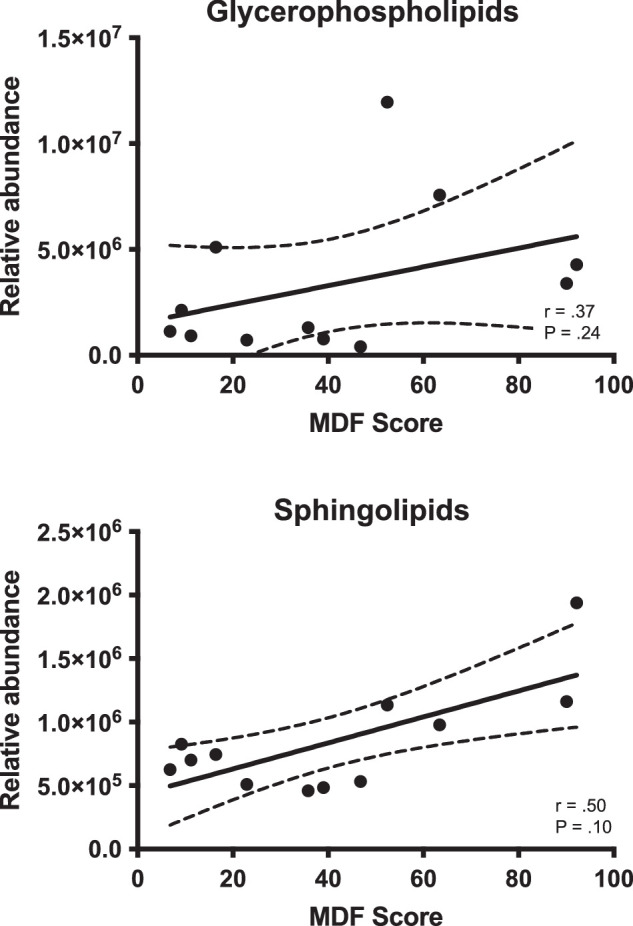
Polar lipid abundance versus MDF scores. Correlation between MDF score and relative abundance of glycerophospholipids and sphingolipids. Neither class showed a correlation to increasing MDF.

The glycerophospholipids were the most abundant class of lipids detected in the tear fluid samples, comprising almost 90% of lipids in the non-MDF samples and 58% in the MDF group (Fig. 2A). These were mostly PC lipids (Fig. 2B), which comprised 88% of lipids in the non-MDF and 50% in the MDF samples. PEs encompassed the remainder of the glycerophospholipids, 8% of lipids in the MDF samples and 2% in the non-MDF samples. Despite being highly abundant in all samples, no correlations were observed between PC (*r* = .37; *P* = 0.24) or PE (*r* = .16; *P* = 0.62) and MDF (Fig. 4). Sphingolipids, primarily sphingomyelin (SM), comprised about 4% of total lipids in the MDF samples and 7% in the non-MDF samples, and were not correlated to MDF (*r* = .50; *P* = 0.10) (Fig. 4).

## Discussion

This study confirms the presence of several lipid classes and species in the FR during SL wear and suggests that nonpolar hydrophobic lipids, specifically WEs, CEs, and TAGs, contribute to MDF. The additional detection of lipids common to cell membranes—namely PC, PE, and SM—suggests that cellular debris exists in the FR independent of MDF severity.

There is no universal method for quantifying MDF, although other techniques have been reported. Carracedo et al.^34^ measured particles per mm^2^ from OCT images, rather than net gray value as was done here, and found that FR turbidity increased by a factor of 8 after 6 to 9 hours of SL wear. Schornack and Nau^33^ measured Scheimpflug tomography images and found a doubling of optical density from 5% to 10% after 2 hours of SL wear. Neither of these studies assessed the FR composition or specified clinically significant MDF, but both are consistent with the findings here that show a range of FR turbidity after several hours of SL wear. The method used to quantify MDF in this study was shown to be highly repeatable both within and between observers and illustrates that MDF is not binary but rather exists as a continuous variable of severity but always a nonzero value. Using a quantitative scale, rather than the more common strategy of categorical assignment,^51^^,^^52^^,^^54^^,^^75^ allows more precision when grading MDF and could be useful in future studies. However, as seen here, it is also helpful to group participants into MDF and non-MDF for some analyses, in which case determining a cutoff may be appropriate.

Lipids across five major lipid categories were detected in the FR samples. The WEs, a class of nonpolar fatty acyl lipids that are primarily secreted by the Meibomian glands,^41^^,^^44^^,^^76^^–^^78^ showed the greatest correlations to MDF. These normally comprise between 13% and 68% of total lipids in the natural tear film,^41^^,^^42^^,^^76^^,^^79^ are characterized by long fatty acid and fatty alcohol chains, and are known for their hydrophobic properties.^43^^–^^47^^,^^78^^,^^80^^–^^84^ In their pure state, they have melting points above physiologic temperatures that increase with each additional carbon atom,^71^^,^^85^ which could explain why longer chain WEs (i.e., >40 carbons) were most significantly increased in MDF. In vivo, the WEs interact with other lipids (i.e., CEs) to lower their melting point and facilitate transparency,^70^^,^^71^ a feature that may be altered when a SL is applied to the eye with aqueous-based (e.g., saline) solutions.

Several species of CEs, which comprise 8% to 39% of the total natural tear lipids,^41^^,^^42^^,^^76^^,^^79^ were elevated in MDF. Like WEs, they are nonpolar, hydrophobic, and primarily secreted by Meibomian glands.^76^^,^^77^^,^^86^ They have a tendency to change phase in aqueous solution,^44^ existing as a solid, a liquid, or an intermediate crystalline state, the latter of which they appear turbid rather than clear.^69^ This state can be affected by temperature, pressure, and interactions with other lipids and surrounding fluid,^69^^,^^71^ so it is plausible that the physical characteristics of the FR change the state of CEs in the FR, contributing to the turbidity observed during MDF.

The TAGs, which are typically the third most abundant nonpolar lipid in the tear film (after WEs and CEs^87^^,^^88^), comprising between 0.05% and 6% of total tear lipids,^41^^,^^42^^,^^76^^,^^79^ were increased in MDF and specifically in the unsaturated form, consistent with the findings of mostly unsaturated TAGs in the natural tear film.^76^^,^^89^ Similar to WEs and CEs, although not as abundant and less understood functionally, the TAGs are believed to contribute to the hydrophobic properties and reduce evaporative loss of the tear film.^44^^,^^88^ Collectively, the correlations of these three classes of hydrophobic nonpolar lipids to MDF provide logical evidence of an alteration in physical form, or dissociation of nonpolar lipids, that creates the turbidity in the aqueous FR observed in MDF.

Polar lipids are far less abundant than nonpolar lipids in the natural tear film, ranging from <1% to 16% of total tear film lipids.^41^^,^^42^^,^^76^^,^^77^^,^^90^^–^^92^ Their proposed functionality is to act as an interface between nonpolar lipids and aqueous tears,^74^^,^^83^^,^^90^^,^^93^ helping to maintain the structure of the tear film.^76^^,^^79^ Although no polar lipids were correlated to MDF, they were present in a greater proportion than expected. For example, PC lipids, which comprise about 0.015% to 0.2% of total natural tear lipids,^44^^,^^77^^,^^89^^,^^90^ were 50% to 90% of total lipids detected in the FR. The PE and SM have likewise been found in variable low concentrations or not at all in natural tears and meibum^87^^,^^89^^,^^90^^,^^92^^,^^94^ but collectively comprised over 10% of the lipids in the FR. The most likely origin of these polar lipids is from cellular debris, as these are common lipids found in cell membranes.^80^^,^^95^ Possible cellular origins are corneal epithelial or perilimbal conjunctival cells, as well as immune cells, which are abundant in the tear film in the morning^96^^–^^101^ and could become trapped when the SL is applied. Since polar lipids are by nature hydrophilic, they likely behave more miscibly with the aqueous FR and are not found to contribute to MDF. There was, however, a trend toward greater SM in MDF that could indicate that there is some aspect of cellular debris that could contribute to MDF, which future studies with larger sample sizes could investigate.

The findings of this study can inform the clinical management of MDF. The Meibomian glands produce most of the lipids in the tears, specifically the nonpolar WEs, CEs, and TAGs that were increased in MDF, so it is important to consider them specifically. Many ocular surface diseases—namely, dry eye diseases such as Meibomian gland dysfunction (MGD)—have altered lipid composition.^86^^,^^89^^,^^102^^–^^106^ Anecdotally, clinicians have linked the occurrence of MDF to patients with dry eye and MGD, and several studies have found alterations in WE and CE lipids in patients with MGD.^86^^,^^104^^,^^107^ This association could suggest that patients with MGD may have a lipid profile that is susceptible to developing MDF. One limitation of this study is that lipid profiles were not evaluated prior to any SL wear, which could be done in future studies to understand if people with altered proportions of select lipids are more susceptible to MDF. Clinically, MGD and other eyelid inflammatory conditions (e.g., blepharitis) should be evaluated and treated accordingly in patients experiencing MDF with SL wear.

There are no strong evidence-based guidelines for managing the SL fit in MDF; however, practitioners will often make several modifications to minimize MDF. Postnikoff et al.^51^ found that greater lens vault was a risk factor for MDF, but the impact of reducing vault or any other SL parameters (i.e., diameter, limbal curves) has yet to be explored in MDF. The SLs in this study were fit similarly on each eye; therefore, no conclusions can be drawn on whether lens modifications could reduce MDF, although there was a greater proportion of eyes with excessive limbal clearance in the MDF group (57%) compared to the non-MDF group (11%). The impact of tear exchange, although it is expected to be minimal,^35^^,^^48^^,^^49^^,^^108^ was not explored here but could certainly impact the FR composition and would be a meaningful future investigation.

Another approach that practitioners employ to reduce MDF is changing the lens application solution,^28^^,^^109^^,^^110^ often to a more viscous solution (i.e., preservative-free artificial tears), although this has not been studied. The nonpolar nature of the lipids correlated to MDF suggests that using an application solution that is more lipophilic (e.g., artificial tear solutions) could reduce the precipitation of nonpolar lipids into the FR. Current SL application solutions approved by the Food and Drug Administration are all aqueous saline-based solutions, so future studies may consider evaluating the use of preservative-free formulations such as artificial tears with nonpolar components as application solutions to reduce MDF during SL wear.

To our knowledge, this is the first study to analyze lipids in the FR during SL wear and can be used to improve future study designs. This was a pilot study with a small sample size, which should be expanded in future studies to include more subjects with a broader range of MDF, perhaps with a deeper investigation to some of the nonpolar lipids found here. Future studies could also attempt to differentiate MDF beyond level of grayscale turbidity, as the clinical presentation of MDF can vary in appearance, sometimes looking more milky and white, other times having a darker, browner hue.^109^ Studies could also consider additional factors that could contribute to MDF, such as differences in participant behavior when applying their lenses (e.g., amount of force applied, whether lid interaction occurred), as this was not specifically controlled here.

In addition to lipids in the FR, soluble proteins, metabolites, and cells in the fluid could contribute to MDF. We previously evaluated cytokines in the FR^32^ but not specifically in MDF, and leukocytes have been detected in both MDF and non-MDF,^51^ but additional proteins or further specification of the cell types in the FR are lacking. We have collected MDF samples that indicate sloughed corneal epithelial cells are also present in the FR (unpublished data), but it is unclear if they contribute to MDF. Future studies should evaluate these additional components of the FR that could be contributory to MDF and assess interventions such as SL modifications or application solutions to determine the best way to mitigate MDF during SL wear.

## Conclusions

The findings of this study suggest that nonpolar lipids—namely, WEs, CEs, and TAGs—contribute to MDF. This does not rule out the presence of other contributors such as cells, proteins, metabolites, or debris. Futures studies should explore the contribution of these other analytes and investigate the use of alternate application solutions to reduce the severity of MDF during SL wear.

## Supplementary Material

Supplement 1

## References

[1] Shorter E, Harthan J, Nau CB, et al. Scleral lenses in the management of corneal irregularity and ocular surface disease. *Eye Cont Lens*. 2018; 44(6): 372–378. 10.1097/ICL.0000000000000436 28968300

[2] Ozek D, Kemer OE, Altiaylik P. Visual performance of scleral lenses and their impact on quality of life in patients with irregular corneas. *Arq Bras Oftalmol*. 2018; 81(6): 475–480. 30231157 10.5935/0004-2749.20180089

[3] Lee JC, Chiu GB, Bach D, Bababeygy SR, Irvine J, Heur M. Functional and visual improvement with prosthetic replacement of the ocular surface ecosystem scleral lenses for irregular corneas. *Cornea*. 2013; 32(12): 1540–1543. 24145631 10.1097/ICO.0b013e3182a73802

[4] Romero-Jiménez M, Flores-Rodríguez P. Utility of a semi-scleral contact lens design in the management of the irregular cornea. *Cont Lens Ant Eye**.* 2013; 36(3): 146–150. 10.1016/j.clae.2012.12.006 23291263

[5] Schornack M, Patel S. Scleral lenses in the management of keratoconus. *Eye Cont Lens*. 2010; 36(1): 39–44. 10.1097/ICL.0b013e3181c786a6 20009945

[6] Fuller D, Wang Y. Safety and efficacy of scleral lenses for keratoconus. *Optom Vis Sci*. 2020; 97(9): 741–748. 32932400 10.1097/OPX.0000000000001578 PMC7547898

[7] Dalton K, Sorbara L. Fitting an MSD (mini scleral design) rigid contact lens in advanced keratoconus with INTACS. *Cont Lens Ant Eye*. 2011; 34(6): 274–281. 10.1016/j.clae.2011.05.001 21664856

[8] Wiwanitkit V. Scleral lenses and ocular cicatricial pemphigoid. *Cornea*. 2010; 29(11): 1330. 20697280 10.1097/ICO.0b013e3181d3fe83

[9] Schornack MM, Pyle J, Patel SV. Scleral lenses in the management of ocular surface disease. *Ophthalmology*. 2014; 121(7): 1398–1405. 24630687 10.1016/j.ophtha.2014.01.028

[10] Schornack MM, Baratz KH. Ocular cicatricial pemphigoid: the role of scleral lenses in disease management. *Cornea*. 2009; 28(10): 1170–1172. 19770718 10.1097/ICO.0b013e318199fa56

[11] Harthan J, Shorter E. Therapeutic uses of scleral contact lenses for ocular surface disease: patient selection and special considerations. *Clin Optom (Auckl)*. 2018; 10: 65–74. 30319297 10.2147/OPTO.S144357 PMC6181806

[12] Kok JH, Visser R. Treatment of ocular surface disorders and dry eyes with high gas-permeable scleral lenses. *Cornea*. 1992; 11(6): 518–522. 1468214 10.1097/00003226-199211000-00006

[13] Romero-Rangel T, Stavrou P, Cotter J, Rosenthal P, Baltatzis S, Stephen FC. Gas-permeable scleral contact lens therapy in ocular surface disease. *Am J Ophthalmol*. 2000; 130(1): 25–32. 11004256 10.1016/s0002-9394(00)00378-0

[14] Rosenthal P, Cotter J. The Boston scleral lens in the management of severe ocular surface disease. *Ophthalmol Clin North Am*. 2003; 16(1): 89–93. 12683251 10.1016/s0896-1549(02)00067-6

[15] Heur M, Bach D, Theophanous C, Chiu GB. Prosthetic replacement of the ocular surface ecosystem scleral lens therapy for patients with ocular symptoms of chronic Stevens-Johnson syndrome. *Am J Ophthalmol*. 2014; 158(1): 49–54. 24699156 10.1016/j.ajo.2014.03.012

[16] Dimit R, Gire A, Pflugfelder SC, Bergmanson JPG. Patient ocular conditions and clinical outcomes using a PROSE scleral device. *Cont Lens Ant Eye*. 2013; 36(4): 159–163. 10.1016/j.clae.2013.02.004 23499361

[17] Rosenthal P, Croteau A. Fluid-ventilated, gas-permeable scleral contact lens is an effective option for managing severe ocular surface disease and many corneal disorders that would otherwise require penetrating keratoplasty. *Eye Cont Lens*. 2005; 31(3): 130–134. 10.1097/01.icl.0000152492.98553.8d 15894881

[18] Koppen C, Kreps EO, Anthonissen L, van Hoey M, Dhubhghaill SN, Vermeulen L. Scleral lenses reduce the need for corneal transplants in severe keratoconus. *Am J Ophthalmol*. 2018; 185: 43–47. 29103959 10.1016/j.ajo.2017.10.022

[19] Bergmanson JP, Walker MK, Johnson LA. Assessing scleral contact lens satisfaction in a keratoconus population. *Optom Vis Sci*. 2016; 93(8): 855–860. 27232897 10.1097/OPX.0000000000000882

[20] Tan DT, Pullum KW, Buckley RJ. Medical applications of scleral contact lenses: 2. gas-permeable scleral contact lenses. *Cornea*. 1995; 14(2): 130–137. 7743793

[21] Visser ES, van der Linden BJ, Otten HM, van der Lelij A, Visser R. Medical applications and outcomes of bitangential scleral lenses. *Optom Vis Sci*. 2013; 90(10): 1078–1085. 23974663 10.1097/OPX.0000000000000018

[22] Pullum KW, Whiting MA, Buckley RJ. Scleral contact lenses: the expanding role. *Cornea*. 2005; 24(3): 269–277. 15778597 10.1097/01.ico.0000148311.94180.6b

[23] Pecego M, Barnett M, Mannis MJ, Durbin-Johnson B. Jupiter scleral lenses: the UC Davis Eye center experience. *Eye Cont Lens*. 2012; 38(3): 179–182. 10.1097/ICL.0b013e31824daa5e 22543730

[24] Tan DDT, Pullum KWK, Buckley RJ. Medical applications of scleral contact lenses: 1. A retrospective analysis of 343 cases. *Cornea*. 1995; 14(2): 130–137. 7743792

[25] Barnett M, Lien V, Li JY, Durbin-Johnson B, Mannis MJ. Use of scleral lenses and miniscleral lenses after penetrating keratoplasty. *Eye Cont Lens*. 2016; 42(3): 185–189. 10.1097/ICL.0000000000000163 26214530

[26] Woods CA, Efron N, Morgan P. Are eye-care practitioners fitting scleral contact lenses? *Clin Exp Optom*. 2020; 103(4): 449–453. 32519339 10.1111/cxo.13105

[27] Barnett M, Ross J, Durbin-Johnson B. Preliminary clinical exploration of scleral lens performance on normal eyes. *J Cont Lens Res Sci*. 2018; 2(2): e14–e21.

[28] Walker MK., Bergmanson J, Miller W, Marsack J, Johnson L. Complications fitting challenges associated with scleral contact lenses: a review. *Cont Lens Ant Eye*. 2016; 39(2): 88–96. 10.1016/j.clae.2015.08.003 26341076

[29] Fadel D. Scleral lens issues and complications related to a non-optimal fitting relationship between the lens and ocular surface. *Eye Cont Lens**.* 2019; 45(3): 152–163. 10.1097/ICL.0000000000000523 29944502

[30] Fisher D, Collins MJ, Vincent SJ. Conjunctival prolapse during open eye scleral lens wear. *Cont Lens Ant Eye*. 2021; 44(1): 115–119. 10.1016/j.clae.2020.09.001 33012674

[31] Courey C, Courey G, Michaud L. Conjunctival inlapse: nasal and temporal conjuctival shape variations associated with scleral lens wear. *J Cont Lens Res Sci*. 2018; 2(1): e–38.

[32] Walker MK, Lema C, Redfern R, Lema C. Scleral lens wear: Measuring inflammation in the fluid reservoir. *Cont Lens Ant Eye*. 2020; 43(6): 577–584. 10.1016/j.clae.2020.02.017 PMC11970577 32165121

[33] Schornack MM, Nau CB. Changes in optical density of postlens fluid reservoir during 2 hours of scleral lens wear. *Eye Cont Lens*. 2018; 44(suppl 2): S344–S349. 10.1097/ICL.0000000000000500 29554027

[34] Carracedo G, Serramito-Blanco M, Martin-Gil A, Wang Z, Rodriguez-Pomar C, Pintor J. Post-lens tear turbidity and visual quality after scleral lens wear. *Clin Exp Optom*. 2017; 100(6): 577. 28125853 10.1111/cxo.12512

[35] Tan B, Zhou Y, Yuen TL, Lin K, Michaud L, Lin MC. Effects of scleral-lens tear clearance on corneal edema and post-lens tear dynamics: a pilot study. *Optom Vis Sci*. 2018; 95(6): 481–490. 29787488 10.1097/OPX.0000000000001220

[36] Fadel D, Toabe M. Scleral lens issues and complications related to handling, care and compliance. *J Cont Lens Res Sci*. 2018; 2(2): e1–e13.

[37] Rathi VM, Mandathara PS, Dumpati S, Sangwan VS. Change in vault during scleral lens trials assessed with anterior segment optical coherence tomography. *Cont Lens Ant Eye*. 2017; 40(3): 157–161. 10.1016/j.clae.2017.03.008 28366677

[38] Sonsino J, Mathe DS. Central vault in dry eye patients successfully wearing scleral lens. *Optom Vis Sci*. 2013; 90(9): e248–e251. 23958714 10.1097/OPX.0000000000000013

[39] Singh S, Basu S. The human lacrimal gland: historical perspectives, current understanding, and 501 recent advances. *Curr Eye Res*. 2020; 45(10): 1188–1198. 32450044 10.1080/02713683.2020.1774065

[40] Pflugfelder SC, Stern ME. Biological functions of tear film. *Exp Eye Res*. 2020; 197: 108115. 32561483 10.1016/j.exer.2020.108115 PMC7483968

[41] Nicolaides N, Kaitaranta JK, Rawdah TN, et al. Meibomian gland studies: comparison of steer and human lipids. *Invest Ophthalmol Vis Sci**.* 1981; 20(4): 522–536. 7194326

[42] Tiffany JM. Individual variations in human Meibomian lipid composition. *Exp Eye Res*. 1978; 27: 289–300. 710541 10.1016/0014-4835(78)90164-1

[43] Butovich IA. On the lipid composition of human meibum and tears: comparative analysis of nonpolar lipids. *Invest Ophthalmol Vis Sci*. 2008; 49(9): 3779–3789. 18487374 10.1167/iovs.08-1889 PMC2659562

[44] Butovich IA. Tear film lipids. *Exp Eye Res*. 2013; 117: 4–27. 23769846 10.1016/j.exer.2013.05.010 PMC3844095

[45] Pucker AD, Nichols JJ. Analysis of meibum and tear lipids. *Ocul Surf*. 2012; 10(4): 230–250. 23084145 10.1016/j.jtos.2012.07.004

[46] Rantamaki AH, Seppanen-Laakso T, Oresic M, et al. Human tear fluid lipidome: from composition to function. *PLoS One*. 2011; 6(5): e19553. 21573170 10.1371/journal.pone.0019553 PMC3088682

[47] Bron AJ, Tiffany JM, Gouveia SM, Yokoi N, Voon LW. Functional aspects of the tear film lipid layer. *Exp Eye Res*. 2004; 78(3): 347–360. 15106912 10.1016/j.exer.2003.09.019

[48] Paugh JR, Chen E, Heinrich C, et al. Silicone hydrogel and rigid gas-permeable scleral lens tear exchange. *Eye Cont Lens*. 2018; 44(2): 97–101. 10.1097/ICL.0000000000000400 29369227

[49] Tse V, Tan B, Kim YH, Zhou Y, Lin MC. Tear dynamics under scleral lenses. *Cont Lens Ant Eye*. 2019; 42(1): 43–48. 10.1016/j.clae.2018.11.016 30545775

[50] Caroline P, Andre M. How much tear exchange occurs beneath scleral lenses. *Cont Lens Spect*. 2014; 29: 64.

[51] Postnikoff CK, Pucker AD, Laurent J, Huisingh C, McGwin G, Nichols JJ. Identification of leukocytes associated with midday fogging in the post-lens tear film of scleral contact lens wearers. *Invest Ophthalmol Vis Sci*. 2019; 60(1): 226–233. 30646011 10.1167/iovs.18-24664 PMC6340400

[52] Schornack MM, Fogt J, Harthan J, et al. Factors associated with patient-reported midday fogging in established scleral lens wearers. *Cont Lens Ant Eye*. 2020; 43(6): 602–608. 10.1016/j.clae.2020.03.005 32201055

[53] Pucker AD, Bickle KM, Jones-Jordan LA, et al. Assessment of a practitioner's perception of scleral contact lens complications. *Cont Lens Ant Eye**.* 2019; 42(1): 15–19. 10.1016/j.clae.2018.11.003 30455084

[54] Skidmore KV, Walker MK, Marsack JD, Bergmanson JPG, Miller WL. A measure of tear inflow in habitual scleral lens wearers with and without midday fogging. *Cont Lens Ant Eye*. 2019; 42(1): 36–42. 10.1016/j.clae.2018.10.009 PMC6340712 30455083

[55] Barnett M. Foggy with no chance of moisture. *Rev Corn Cont Lens*. 2018; May/June: 16–19.

[56] Caroline PP, Andre M. Cloudy vision with sclerals. *Cont Lens Spec*. 2012; 27: 56.

[57] Walker MK, Morrison S, Caroline PJ, et al. Laboratory analysis of scleral lens tear reservoir clouding. In: *Global Specialty Lens Symposium; Las Vegas, NV*. 2014.

[58] Chen J, Keirsey JK, Green KB, Nichols KK. Expression profiling of nonpolar lipids in meibum from patients with dry eye: a pilot study. *Invest Ophthalmol Vis Sci*. 2017; 58: 2266–2274. 28426869 10.1167/iovs.16-20902 PMC5398788

[59] Chen J, Green KB, Nichols KK. Compositional analysis of wax esters in human meibomian 541 gland secretions by direct infusion electrospray ionization mass spectrometry. *Lipids*. 2016; 51(11): 1269–1287. 27571784 10.1007/s11745-016-4183-4 PMC5075507

[60] Terry R, Schnider C, Holden B, et al. CCLRU standards for success of daily and extended wear contact lenses. *Optom Vis Sci*. 1993; 70(3): 234–243. 8483586 10.1097/00006324-199303000-00011

[61] Bartlett JW, Frost C. Reliability, repeatability and reproducibility: analysis of measurement errors in continuous variables. *Ultrasound Obstet Gynecol*. 2008; 31(4): 466–475. 18306169 10.1002/uog.5256

[62] Bland JM, Altman DG. Measurement error. *Br Med J*. 1996; 313(7059): 744. 8819450 10.1136/bmj.313.7059.744 PMC2352101

[63] Bailey L, Prajapati D, Basso K. Optimization of the sulfo-phospho-vanillin assay for sample normalization in unlabeled quantitative lipidomic LC-MS/MS. *Anal Chem*. 2022; 94(51): 17810–17818. 36520113 10.1021/acs.analchem.2c03488

[64] Kind T, Liu KH, Lee DY, Defelice B, Meissen JK, Fiehn O. Lipidblast in silico tandem mass spectrometry database for lipid identification. *Nat Methods*. 2013; 10(8): 755. 23817071 10.1038/nmeth.2551 PMC3731409

[65] Tsugawa H, Ikeda K, Takahashi M, et al. A lipidome atlas in MS-DIAL 4. *Nat Biotechnol*. 2020; 38: 1159–1163. 32541957 10.1038/s41587-020-0531-2

[66] Kothalawala N, Mudalige TK, Sisco P, Linder SW. Novel analytical methods to assess the chemical and physical properties of liposomes. *J Chromatogr B Analyt Technol Biomed Life Sci*. 2018; 1091: 14–20. 10.1016/j.jchromb.2018.05.028 29803685

[67] Sud M, Fahy E, Cotter D, et al. LMSD: LIPID MAPS structure database. *Nucleic Acids Res*. 2007; 35(Database): D527–D532. 17098933 10.1093/nar/gkl838 PMC1669719

[68] Fahy E, Sud M, Cotter D, Subramaniam S. LIPID MAPS online tools for lipid research. *Nucleic Acids Res*. 2007; 35(Web Server): W606–W612. 17584797 10.1093/nar/gkm324 PMC1933166

[69] Ginsburg GS, Atkinson D, Small DM. Physical properties of cholesteryl esters. *Prog Lipid Res*. 1984; 23: 135–167. 6399750 10.1016/0163-7827(84)90002-x

[70] Butovich IA. Lipidomics of human Meibomian gland secretions: chemistry, biophysics, and physiological role of Meibomian lipids. *Prog Lipid Res*. 2011; 50(3): 278–301. 21458488 10.1016/j.plipres.2011.03.003 PMC3114158

[71] Patel N, Dennis R, Gibbs AG. Chemical and physical analyses of wax ester properties. *J Insect Sci*. 2001; 1(4): 1–7. 15455064 10.1673/031.001.0401 PMC355888

[72] Kulovesi P, Rantamäki AH, Holopainen JM, Holopai-Nen JM. Surface properties of artificial 568 tear film lipid layers: effects of wax esters. *Invest Ophthalmol Vis Sci*. 2014; 55: 4448–4454. 24876287 10.1167/iovs.14-14122

[73] Borchman D, Ramasubramanian A, Foulks GN. Human meibum cholesteryl and wax ester variability with age, sex, and meibomian gland dysfunction. *Invest Ophthalmol Vis Sci*. 2019; 60(6): 2286–2293. 31112994 10.1167/iovs.19-26812 PMC6530518

[74] Korb D, Greiner J, Glonek T. Tear film lipid layer formation: implications for contact lens wear. *Optom Vis Sci*. 1996; 73(3): 189–192. 8725021 10.1097/00006324-199603000-00011

[75] Fogt JS, Karres M, Barr JT. Changes in symptoms of midday fogging with a novel scleral contact lens filling solution. *Optom Vis Sci*. 2020; 97(9): 690–696. 32941340 10.1097/OPX.0000000000001559 PMC7547899

[76] Chen J, Green-Church KB, Nichols KK. Shotgun lipidomic analysis of human meibomian gland secretions with electrospray ionization tandem mass spectrometry. *Invest Ophthalmol Vis Sci*. 2010; 51(12): 6220–6231. 20671273 10.1167/iovs.10-5687 PMC3055753

[77] Butovich IA, Uchiyama E, di Pascuale MA, McCulley JP. Liquid chromatography-mass spectrometric analysis of lipids present in human meibomian gland secretions. *Lipids*. 2007; 42(8): 765–776. 17605062 10.1007/s11745-007-3080-2

[78] Butovich IA, Wojtowicz JC, Molai M. Human tear film and meibum: very long chain wax esters and (O-acyl)-omega-hydroxy fatty acids of meibum. *J Lipid Res*. 2009; 50(12): 2471–2485. 19535818 10.1194/jlr.M900252-JLR200 PMC2781319

[79] Mcculley JP, Shine W. A compositional based model for the tear film lipid layer. *Tr Am Ophth Soc*. 1997; XCV: 79–93. PMC1298352 9440164

[80] Nagyová B, Tiffany JM. Components responsible for the surface tension of human tears. *Curr Eye Res*. 1999; 19(1): 4–11. 10415451 10.1076/ceyr.19.1.4.5341

[81] Berger RE, Corrsin S. A surface tension gradient mechanism for driving the pre-corneal tear film after a blink. *J Biomech*. 1974; 7(3): 225–238. 4846263 10.1016/0021-9290(74)90013-x

[82] Fahy E, Cotter D, Sud M, Subramaniam S. Lipid classifications, structures and tools. *Biochim Biophys Acta*. 2011; 1811(11): 637–647. 21704189 10.1016/j.bbalip.2011.06.009 PMC3995129

[83] Wizert A, Robert Iskander D, Cwiklik L, Roccatano D. Organization of lipids in the tear film: a molecular-level view. *PLoS One*. 2014; 9(3): e92461. 24651175 10.1371/journal.pone.0092461 PMC3961367

[84] Köfeler HC. Branched fatty acids. In: Wenk MR, ed. *Encyclopedia of Lipidomics*. Dordrecht: Springer; 2016: 1–3.

[85] Iyengar B, Schlenk H. Melting points of synthetic wax esters. *Lipids*. 1969; 4(1): 28–30. 5766845 10.1007/BF02531790

[86] Borchman D, Ramasubramanian A, Foulks GN, Boulevard MA. Human meibum cholesteryl and wax ester variability with age, sex, and meibomian gland dysfunction. *Invest Ophthalmol Vis Sci**.* 2019; 60(6): 2286–2293. 31112994 10.1167/iovs.19-26812 PMC6530518

[87] Brown SHJ, Kunnen CME, Duchoslav E, et al. A comparison of patient matched meibum and tear lipidomes. *Invest Ophthalmol Vis Sci*. 2013; 54(12): 7417–7423. 24135754 10.1167/iovs.13-12916

[88] Masoudi S, Mitchell TW, Willcox MD. Profiling of non-polar lipids in tears of contact lens wearers during the day. *Exp Eye Res*. 2021; 207: 108567. 33848523 10.1016/j.exer.2021.108567

[89] Lam SM, Tong L, Yong SS, et al. Meibum lipid composition in Asians with dry eye disease. *PLoS One*. 2011; 6(10): e24339. 22043274 10.1371/journal.pone.0024339 PMC3197196

[90] Shine WE, Mcculley JP. Polar lipids in human meibomian gland secretions. *Curr Eye Res*. 2003; 26(2): 89–94. 12815527 10.1076/ceyr.26.2.89.14515

[91] Shine WE, Mcculley JP. Meibomianitis polar lipid abnormalities. *Cornea*. 2004; 23(8): 781–783. 15502478 10.1097/01.ico.0000133995.99520.1f

[92] Butovich IA, Uchiyama E, McCulley JP. Lipids of human meibum: mass-spectrometric analysis and structural elucidation. *J Lipid Res*. 2007; 48(10): 2220–2235. 17626978 10.1194/jlr.M700237-JLR200

[93] Telenius J, Koivuniemi A, Kulovesi P, Holopainen JM, Vattulainen I. Role of neutral lipids in tear fluid lipid layer: coarse-grained simulation study. *Langmuir*. 2012; 28(49): 17092–17100. 23151187 10.1021/la304366d

[94] Gao H, Chen H, Xie HT, Xu KK, Shi BJ, Huang YK. Changes in meibum lipid composition with ocular demodex infestation. *Transl Vis Sci Technol*. 2021; 10(14): 1–9. 10.1167/tvst.10.14.6 PMC8662575 34874449

[95] Dean AW, Glasgow BJ. Mass spectrometric identification of phospholipids in human tears and tear lipocalin. *Invest Ophthalmol Vis Sci*. 2012; 53(4): 1773–1782. 22395887 10.1167/iovs.11-9419 PMC3342792

[96] Gorbet M, Postnikoff C, Williams S. The noninflammatory phenotype of neutrophils from the closed-eye environment: a flow cytometry analysis of receptor expression. *Invest Ophthalmol Vis Sci*. 2015; 56(8): 4582–4591. 26200498 10.1167/iovs.14-15750

[97] Postnikoff CK, Held K, Viswanath V, Nichols KK. Enhanced closed eye neutrophil degranulation in dry eye disease. *Ocul Surf*. 2020; 18(4): 841–851. 32889089 10.1016/j.jtos.2020.08.011

[98] Wilson G, O'Leary D, Holden B. Cell content of tears following overnight wear of a contact lens. *Curr Eye Res*. 1989; 8(4): 329–335. 2470550 10.3109/02713688908996380

[99] Tan K, Sack R, Holden B, Swarbrick H. Temporal sequence of changes in tear film composition during sleep. *Curr Eye Res*. 1993; 12(11): 1001–1007. 8306709 10.3109/02713689309029226

[100] Sack RA, Beaton A, Sathe S, Morris C, Willcox M, Bogart B. Towards a closed eye model of the pre-ocular tear layer. *Prog Retin Eye Res*. 2000; 19(6): 649–668. 11029550 10.1016/s1350-9462(00)00006-9

[101] Sack RA, Tan KO, Tan A, Ooi Tan Kah, Tan A. Diurnal tear cycle: evidence for a nocturnal inflammatory constitutive tear fluid. *Invest Ophthalmol Vis Sci*. 1992; 33(3): 626–640. 1544788

[102] Bland HC, Moilanen JA, Ekholm FS, Paananen RO. Investigating the role of specific tear film lipids connected to dry eye syndrome: a study on O-acyl-ω-hydroxy fatty acids and diesters. *Langmuir*. 2019; 35(9): 3545–3552. 30712353 10.1021/acs.langmuir.8b04182

[103] Foulks GN. The correlation between the tear film lipid layer and dry eye disease. *Surv Ophthalmol*. 2007; 52(4): 369–374. 17574063 10.1016/j.survophthal.2007.04.009

[104] Joffre C, Souchier M, Grégoire S, et al. Differences in meibomian fatty acid composition in patients with meibomian gland dysfunction and aqueous-deficient dry eye. *Br J Ophthalmol*. 2008; 92(1): 116–119. 18156378 10.1136/bjo.2007.126144

[105] Hetman ZA, Borchman D. Concentration dependent cholesteryl-ester and wax-ester structural relationships and meibomian gland dysfunction. *Biochem Biophys Rep*. 2020; 21: 100732. 32042930 10.1016/j.bbrep.2020.100732 PMC7000810

[106] Borchman D, Yapper M, Foulks G. Changes in human meibum lipid with meibomian gland dysfunction using principal component analysis. *Exp Eye Res*. 2010; 91(2): 246–256. 20546726 10.1016/j.exer.2010.05.014 PMC2914467

[107] Borchman D, Foulks GN, Yappert MC, Milliner SE. Differences in human meibum lipid composition with meibomian gland dysfunction using NMR and principal component analysis. *Invest Ophthalmol Vis Sci*. 2012; 53(1): 337–347. 22131391 10.1167/iovs.11-8551 PMC3292369

[108] Morrison S, Walker M, Caroline P. Tear exchange beneath scleral lenses? In: *Global Specialty Lens Symposium; Las Vegas, NV*. 2015.

[109] Johns L. Scleral lenses, turbid clouding. *I-site Newsletter*. 2014;November:online, http://www.netherlens.com/home.

[110] Caroline PP, Andre M. Cloudy vision with sclerals. *Cont Lens Spect*. 2012; 27: 56.

