# Supplementary material for: Nonpolar Lipids Contribute to Midday Fogging During Scleral Lens Wear
Source: Invest Ophthalmol Vis Sci. 2023 Jan 11;64(1):7. doi: 10.1167/iovs.64.1.7 (PMC9840443; doi:10.1167/iovs.64.1.7)
Supplement: Supplement 1 [file iovs-64-1-7_s001.pdf]

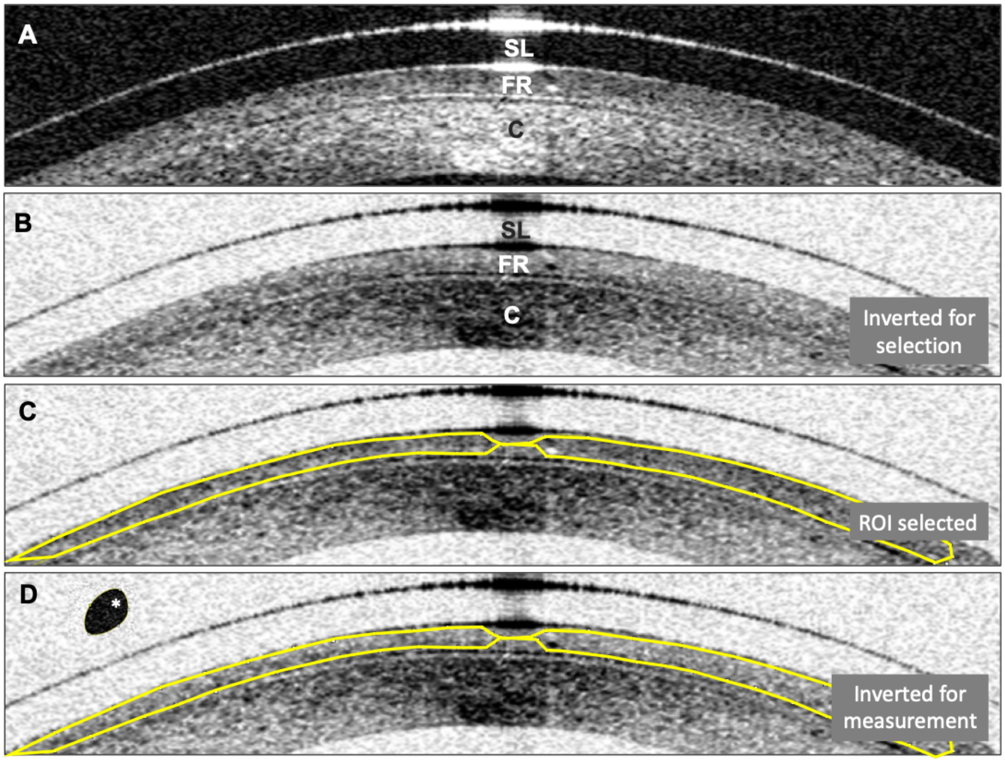

Supplemental Figure 1. AS-OCT Image processing to determine MDF score. First, the raw image was imported into ImageJ (A), and inverted to improve contrast (B). The region of interest (ROI) was selected using the polygon selection tool and splinefit (C), and the entire FR area was selected (yellow outline). Lastly, the selected ROI was inverted again, and the mean gray value was measured in "calibrated optical units" (D). Due to the noise of the images, a small area in the periphery of the imaging screen was randomly selected (D, white star), and mean gray value was measured using the same technique as the ROI, which was later subtracted from the FR value to determine the final score for each image. Scores were measured two times by two different masked examiners and averaged for the final score. C: cornea; FR: fluid reservoir; SL: scleral lens

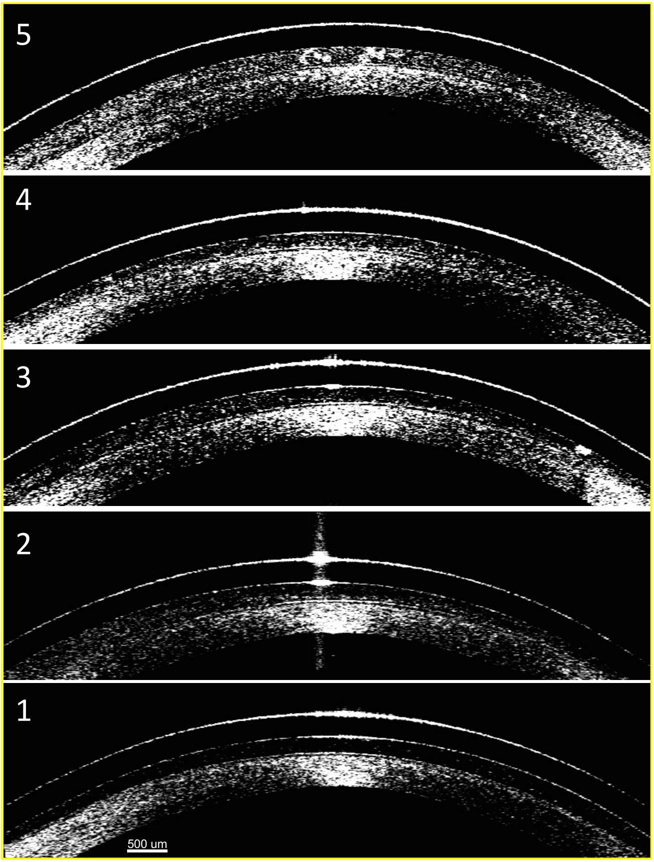

Supplemental Figure 2. Subjective MDF grading scale. The 5-point scale used for subjective grouping of MDF images.

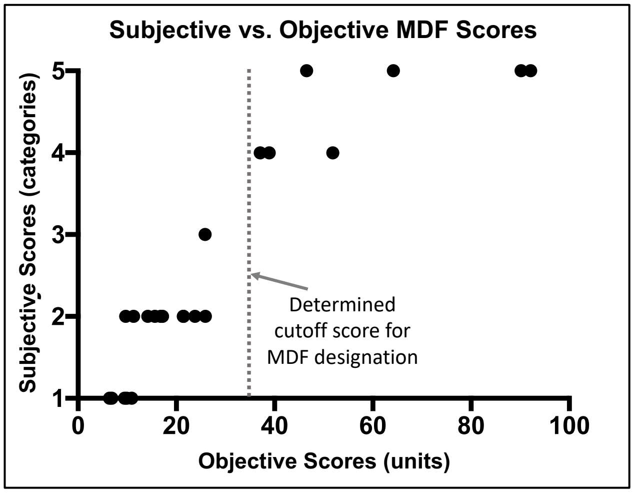

Supplemental Figure 3. Subjective vs. objective MDF. The subjective categories are compared to the objective scores, showing that the subjective scoring agreed with those objective scores. However, the objective scores are more able to discriminate between severity within each of the subjective categories.

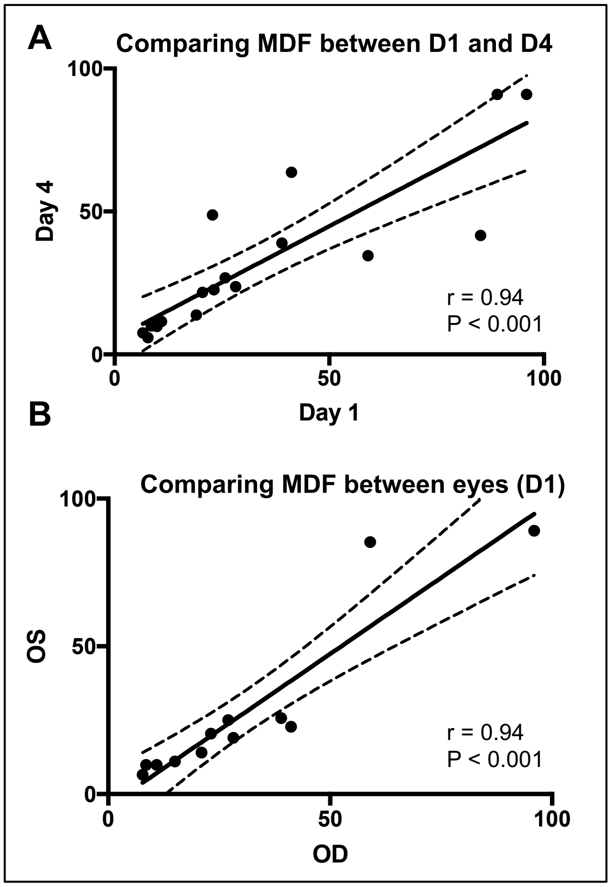

Supplemental Figure 4. MDF score correlations between days and eyes. A comparison of each eyes' MDF scores between day 1 and day 4 (A), and the right versus left eyes on day 1 (B) show strong correlations within and between eyes.

Supplemental Table 1. Raw relative absorbance lipid data

	m/z	Name	Cat.	Class	Formula	04	07	10	11	06	02	05	12	01	08	09	03
G0: MDF; G1: non-MDF						G0	G0	G0	G0	G0	G0	G0	G1	G1	G1	G1	G1
						SPVA	SPVA	SPVA	SPVA	SPVA	SPVA	SPVA	SPVA	SPVA	SPVA	SPVA	SPVA
72.5	608.6319	Behenyl oleate	Sterol	WE	C40.0H78.0O2	175474	196826	265336	430644	52104	115176	90250	70384	41622	91936	206680	119360
72.5	640.6021	CE 16:1	Sterol	CE	C43H74O2	30106	22058	22300	25654	9662	12832	11466	25698	9692	21202	17870	22922
74.1	668.6322	CE 18:1	Sterol	CE	C45H78O2	46554	72740	50764	52182	22844	25110	22168	56496	19114	41222	23570	49264
72.7	666.6166	CE 18:2	Sterol	CE	C45H76O2	574896	371930	224782	129208	46926	82988	180106	577242	65698	567566	87862	665838
71.4	664.6011	CE 18:3	Sterol	CE	C45H74O2	45042	21334	15368	8396	2708	4332	8090	29156	3662	17230	5652	25790
75.9	684.6638	CE 19:0	Sterol	CE	C46H82O2	33286	63114	53770	89076	16942	39724	17334	24686	13074	17794	45944	36488
76.7	698.6789	CE 20:0	Sterol	CE	C47H84O2	188802	188122	266926	483318	58250	186372	72454	85796	53668	92392	202780	148530
75.4	696.6636	CE 20:1	Sterol	CE	C47H82O2	143656	203060	200512	344970	48842	117092	65078	76508	42946	71968	156400	107008
73.1	694.6472	CE 20:2	Sterol	CE	C47H80O2	10004	9278	12590	20582	2752	5266	4468	0	3154	0	9924	6210
72.0	690.6166	CE 20:4	Sterol	CE	C47H76O2	246578	122668	73054	31190	10254	19488	40022	190746	17244	135124	17328	155798
77.6	712.6945	CE 21:0	Sterol	CE	C48H86O2	161772	156414	215390	420204	46442	123828	56850	59584	40070	68960	153130	117374
78.4	726.7102	CE 22:0	Sterol	CE	C49H88O2	157118	159016	220394	438958	50590	153464	59318	65304	45352	74802	167114	120356
77.0	724.6948	CE 22:1	Sterol	CE	C49H86O2	254170	227926	308928	588998	69060	174268	95868	94616	57200	103240	210564	150316
75.7	722.6792	CE 22:2	Sterol	CE	C49H84O2	52980	50520	99206	213822	17174	53050	21868	23254	16712	29186	54268	39492
71.3	714.615	CE 22:6	Sterol	CE	C49H76O2	19750	10358	6192	4136	1906	3168	4140	18542	2996	13600	2352	11332
79.3	740.726	CE 23:0	Sterol	CE	C50H90O2	155050	158818	198598	415000	49198	120616	57268	61254	39392	66274	161236	128492
78.7	752.7259	CE 24:1	Sterol	CE	C51H90O2	451266	402860	523782	1037218	128918	385062	159778	159680	118402	186412	355178	263792
75.9	750.7094	CE 24:2	Sterol	CE	C51H88O2	19482	18566	32276	57960	6192	19124	9680	9206	5314	8882	17420	13552
77.2	750.7103	CE 24:2	Sterol	CE	C51H88O2	35052	35736	63446	130410	10694	35824	14174	17656	12454	17334	30586	26188
65.0	768.7579	CE 25:0	Sterol	CE	C52H94O2	16626	16336	31306	0	14964	0	0	26918	35302	0	17934	0
68.6	782.7727	CE 26:0	Sterol	CE	C53H96O2	0	0	75902	144576	0	109956	3016	0	0	0	0	95000
69.0	782.7727	CE 26:0	Sterol	CE	C53H96O2	29564	51540	73698	0	59166	0	0	70986	140286	0	59968	0
80.0	780.7571	CE 26:1	Sterol	CE	C53H94O2	15282	16300	29770	69016	0	0	8098	0	9572	0	25250	18752
77.5	778.7417	CE 26:2	Sterol	CE	C53H92O2	15406	14868	21194	44406	5706	14704	5888	6438	4958	6236	15410	12008
71.4	796.7882	CE 27:0	Sterol	CE	C54H98O2	23506	49718	75486	152328	50282	128610	0	70956	116176	0	61140	105544
73.1	810.8044	CE 28:0	Sterol	CE	C55H100O2	5348	11904	16192	34388	13754	28350	0	15286	29600	0	12734	20344
69.4	808.7888	CE 28:1	Sterol	CE	C55H98O2	0	42204	71366	99058	19788	80180	0	0	0	0	44878	63338
74.5	824.8191	CE 29:0	Sterol	CE	C56H102O2	9032	16562	22718	43864	18808	46750	0	23198	40214	0	18952	32130
73.4	836.82	CE 30:1	Sterol	CE	C57H102O2	23162	67158	100378	166214	63038	146234	4020	108196	134162	0	62330	100538
76.1	864.8511	CE 32:1	Sterol	CE	C59H106O2	17810	41834	81062	193714	47370	159094	3392	65558	103054	0	58806	94398
76.2	890.8661	CE 34:2	Sterol	CE	C61H108O2	3530	5490	9662	30426	6572	24678	1886	6770	13130	0	6870	10228
73.9	1026.994	CE 44:4	Sterol	CE	C71H124O2	97598	96770	111902	131396	106896	116492	142218	81358	132272	131226	188242	160490
62.0	650.6433	Cer 18:1_2O_24:0	SL	Cer	C42H83NO3	34282	11714	7316	7422	2664	4320	5100	14314	3716	4000	3004	0
69.6	1043.968	DE-ch	PK	Flav	C71H126O4	3942	3128	4406	9042	11286	11210	17796	6936	13346	12394	20832	12310
57.5	614.5702	DG 16:0_18:0	GL	DAG	C37H72O5	16528	11512	11408	10180	9722	12130	10426	11392	12360	12446	13080	9252
73.7	636.6637	Hexacosanyl palmitoleate	Sterol	WE	C42.0H82.0O2.0	356910	422444	566988	874614	113014	287822	141694	171674	87182	185820	285504	215652
10.9	482.3237	LPC 15:0	GP	PC	C23H48NO7P	182734	364502	1509498	705772	399472	310866	150320	272132	362542	498192	245650	509630
38.3	734.5669	PC 16:0_16:0	GP	PC	C40H80NO8P	112140	75588	43684	34440	15162	19186	59306	44546	43252	80480	26116	26206
33.3	732.5525	PC 16:0_16:1	GP	PC	C40H78NO8P	104842	65044	33556	25576	5170	8678	16318	30204	19584	39058	11988	12080
39.1	760.5841	PC 16:0_18:1	GP	PC	C42H82NO8P	1211092	781590	425356	321738	44640	87380	178936	557960	178326	287084	76930	102436
36.3	784.5833	PC 16:0_20:3	GP	PC	C44H82NO8P	425534	255914	126418	83112	6326	19416	18380	165116	13172	50440	12920	15420
30.3	780.5521	PC 16:0_20:5	GP	PC	C44H78NO8P	75448	42070	20578	12890	1138	2320	1670	22292	1544	3512	1586	1428
34.5	808.5839	PC 16:0_22:5	GP	PC	C46H82NO8P	461980	271806	146058	98844	7804	15582	21238	159152	20264	54912	12014	21074
32.6	806.5676	PC 16:0_22:6	GP	PC	C46H80NO8P	301052	172024	85806	60468	3626	9946	7250	111126	6746	30744	4172	8510
29.7	756.5522	PC 16:1_18:2	GP	PC	C42H78NO8P	33938	22296	11140	8588	1326	2058	3998	13062	4486	4430	3356	2454
34.7	758.5681	PC 17:1_17:1	GP	PC	C42H80NO8P	3961628	2593704	1610928	1306440	117852	289900	437694	1663868	322906	689450	296768	317904
45.1	788.6146	PC 18:0_18:1	GP	PC	C44H86NO8P	189880	118324	72326	54476	11778	19100	55430	108246	60768	69722	21426	28550
42.4	812.6144	PC 18:0_20:3	GP	PC	C46H86NO8P	197374	115682	54572	41794	3712	9306	10978	95292	8352	35994	5148	12562
40.4	836.6143	PC 18:0_22:5	GP	PC	C48H86NO8P	50996	28190	15454	11832	1486	3070	3940	24374	2204	8982	0	4480
39.9	786.5994	PC 18:1_18:1	GP	PC	C44H84NO8P	133432	75724	48258	40608	10230	13784	42972	63070	60116	45708	26416	18552
40.7	786.5994	PC 18:1_18:1	GP	PC	C44H84NO8P	1284250	797516	441188	338808	27772	75570	136694	628128	98474	222590	54064	108312
35.4	784.5838	PC 18:1_18:2	GP	PC	C44H82NO8P	319360	184934	111162	99796	16344	29624	70696	126956	80666	64634	37464	33254

31.0	782.5682	PC 18:2_18:2	GP	PC	C44H80NO8P	122804	73948	36690	34700	4034	7920	21780	36746	20720	15566	9746	9996
34.0	782.5681	PC 18:2_18:2	GP	PC	C44H80NO8P	1393538	837702	419260	311096	13462	43690	32700	532426	39346	126856	23494	39622
38.1	810.5985	PC 19:2_19:2	GP	PC	C46H84NO8P	86472	56204	28286	22598	1758	4360	5672	27002	4510	14992	3342	4318
40.0	810.5994	PC 19:2_19:2	GP	PC	C46H84NO8P	830268	497944	252372	195846	10360	36020	40020	387940	28514	118530	15204	41206
44.2	838.6296	PC 20:2_20:2	GP	PC	C48H88NO8P	37544	20580	10950	7892	1192	2204	2606	18142	1536	7450	1038	3034
38.6	834.599	PC 20:3_20:3	GP	PC	C48H84NO8P	91150	56922	31502	20122	1206	4946	5912	48234	4100	15438	1634	4812
10.8	520.3383	PC O-18:2	GP	PC	C26H50NO7P	4414	32798	28844	28620	25300	6692	12602	12072	7252	11758	6992	16000
42.2	720.5872	PC O-32:0	GP	PC	C40H82NO7P	15802	11488	6076	5096	3734	1356	3272	8372	3482	4538	2256	2154
37.7	768.5881	PC O-36:4	GP	PC	C44H82NO7P	114072	68518	33782	23326	2826	4986	6146	42692	4704	15632	0	6686
36.8	766.5729	PC O-36:5	GP	PC	C44H80NO7P	61952	29090	14654	11508	2758	3532	7168	26042	6752	12178	2368	7580
43.9	796.6194	PC O-38:4	GP	PC	C46H86NO7P	41550	27022	13054	9978	1276	2468	3896	20678	2100	7336	1438	3516
38.2	794.6038	PC O-38:5	GP	PC	C46H84NO7P	119718	68418	30194	24946	2528	5138	7634	39770	5896	17354	3148	6716
11.9	522.3551	PC_18:1_6Z_0:0	GP	PC	C26.0H52.0N1.0O7.0P1.0	10714	35596	30360	57612	24622	9026	17398	8568	13072	16550	12530	20394
30.2	806.5678	PC_18:2_20:4	GP	PC	C46.0H80.0N1.0O8.0P1.0	57358	32814	17802	12310	1180	1968	2710	19738	3500	4686	1730	2146
13.8	524.3704	PC_O-16:0_2:0	GP	PC	C26.0H54.0N1.0O7.0P1.0	26706	64482	31624	44302	23502	11892	25090	14836	11274	25532	12188	22740
42.8	744.5523	PE 18:0_18:2	GP	PE	C41H78NO8P	9006	6900	6534	7884	2306	3884	8122	6848	10046	4242	5504	3154
34.5	764.5205	PE 18:1_20:5	GP	PE	C43H74NO8P	21084	14220	9262	7446	2024	3996	3694	11852	5436	3578	4384	2178
44.6	702.5429	PE O-16:1_18:1	GP	PE	C39H76NO7P	6788	6270	7152	8170	3392	4348	12490	5864	14440	5740	8762	4622
39.1	724.5265	PE O-16:1_20:4	GP	PE	C41H74NO7P	14154	13456	11070	14480	7446	7940	17290	8578	22472	5932	13088	5700
73.2	622.6478	Pentacosanyl palmitoleate	Sterol	WE	C41.0H80.0O2.0	266390	308468	376438	645272	85968	152328	120454	102648	58386	130820	283382	184348
40.1	904.589	PI 38:4	GP	PS	C47H83O13P	23346	18526	10158	7694	1420	2624	2650	10160	3924	4156	1848	2770
45.8	790.5595	PS 14:0_22:1	GP	SEP	C42H80NO10P	7396	7356	10598	12506	4538	7726	12714	5452	21566	7442	11950	5132
38.6	731.6043	SM 10:0_2O_26:1	SL	SM	C41H83N2O6P	42020	27628	20224	18584	3504	4974	14238	19810	7972	16508	7532	8360
26.8	675.5429	SM 16:1_2O_16:0	SL	SM	C37H75N2O6P	17800	13992	12084	13862	8750	5278	6362	6596	4604	7632	11300	6016
56.1	815.6984	SM 18:0_2O_24:1	SL	SM	C47H95N2O6P	89020	48700	36280	45384	14382	21442	32114	50382	21486	20142	31410	13174
32.5	703.5735	SM 18:1_2O_16:0	SL	SM	C39H79N2O6P	259036	187204	135730	132010	40114	48294	94740	113674	62366	104790	73272	66286
50.4	813.6828	SM 18:1_2O_24:1	SL	SM	C47H93N2O6P	141224	82220	46750	49978	8330	14394	21368	74778	12640	35078	13722	13518
27.7	701.5576	SM 18:2_2O_16:0	SL	SM	C39H77N2O6P	36386	23332	13712	14420	3938	4176	5610	14792	3288	11980	6792	8342
33.7	729.5885	SM 18:2_2O_18:0	SL	SM	C41H81N2O6P	24582	14846	7652	6486	1356	2160	3810	8980	2020	7296	2410	5554
45.8	811.667	SM 18:2_2O_24:1	SL	SM	C47H91N2O6P	81154	51052	26532	22934	4032	8726	11148	40470	6752	26274	6046	10524
50.7	787.6673	SM 19:0_2O_21:1	SL	SM	C45H91N2O6P	76024	45420	30598	37210	9016	14978	20612	40944	12188	14074	19144	10238
46.1	785.6512	SM 23:1_2O_17:1	SL	SM	C45H89N2O6P	39128	22042	14250	11466	2106	3636	5418	16996	2680	13206	3038	5682
34.8	705.589	SM 24:0_2O_10:0	SL	SM	C39H81N2O6P	33904	33786	38828	35424	11514	13284	45386	21348	81774	34938	44824	18124
35.8	811.6646	SM 29:2_2O_13:1	SL	SM	C47H91N2O6P	11014	12460	0	11974	10602	10342	10182	12754	10216	10628	12154	11002
53.5	801.6825	SM 33:1_2O_8:0	SL	SM	C46H93N2O6P	32286	15466	11966	16854	3384	4762	7434	18184	4576	6048	6198	4048
44.8	759.6363	SM 34:1_2O_4:0	SL	SM	C43H87N2O6P	25564	15506	16108	19078	4308	5816	11808	13316	6522	6890	10658	5020
62.6	684.6117	TG 12:0_12:0_14:0	GL	TAG	C41H78O6	29954	13314	15878	13568	12230	11878	13114	18312	13388	11848	14692	11880
65.3	712.6433	TG 12:0_14:0_14:0	GL	TAG	C43H82O6	32836	16932	17690	19352	15044	16110	15572	18188	16248	12144	18036	13858
67.5	740.6741	TG 12:0_14:0_16:0	GL	TAG	C45H86O6	39080	19632	23206	28084	19570	20058	21044	20058	18696	15764	24120	21096
68.2	754.689	TG 14:0_14:0_15:0	GL	TAG	C46H88O6	24186	15388	19820	23124	13660	15874	14594	16022	14156	12210	24714	15724
69.3	768.7046	TG 14:0_14:0_16:0	GL	TAG	C47H90O6	62756	36548	44598	55890	39786	42604	41446	40898	40126	30270	65224	42856
66.2	764.6743	TG 14:0_14:1_16:1	GL	TAG	C47H86O6	30284	16422	20308	22810	14334	15962	18984	14540	15984	11878	31148	15842
70.0	782.7207	TG 14:0_15:0_16:0	GL	TAG	C48H92O6	64980	41886	52968	66052	41734	51336	46012	42478	44584	35662	73800	46652
68.7	780.7038	TG 14:0_15:1_16:0	GL	TAG	C48H90O6	69002	40256	56260	78778	40812	45638	48300	41506	42436	34954	77188	45932
71.0	796.7357	TG 14:0_16:0_16:0	GL	TAG	C49H94O6	69952	59744	70270	69436	61148	62172	57594	62964	60000	50794	58184	61374
69.5	794.7227	TG 14:0_16:0_16:1	GL	TAG	C49H92O6	153204	100828	133908	176590	68542	121398	121666	105932	111156	90936	189562	114060
68.3	792.7048	TG 14:0_16:1_16:1	GL	TAG	C49H90O6	110800	61146	83218	98486	64352	72664	66806	65522	66688	49088	120728	71408
67.2	778.6911	TG 14:1_15:0_16:1	GL	TAG	C48H88O6	36880	19652	25156	31232	17896	26132	23552	18916	19992	15460	37010	21238
66.7	790.6901	TG 14:1_16:1_16:1	GL	TAG	C49H88O6	28384	14958	17474	19966	14776	16538	16848	16484	15214	11782	25554	16574
71.5	810.7516	TG 15:0_16:0_16:0	GL	TAG	C50H96O6	77270	60158	78950	101216	55664	64174	61966	65548	58816	51676	93510	63020
70.3	808.7363	TG 15:0_16:0_16:1	GL	TAG	C50H94O6	130796	86610	116062	158110	89696	100468	92914	91372	93240	74230	161888	99108
71.7	836.7695	TG 15:0_16:0_18:1	GL	TAG	C52H98O6	81188	71236	100740	139928	64798	80530	72374	70270	68056	57550	117970	75696
76.3	922.8766	TG 15:0_16:0_24:0	GL	TAG	C58H112O6	13896	12412	14752	20346	11968	13686	12300	13482	11652	10390	17594	12744

69.1	806.7217	TG 15:0_16:1_16:1	GL	TAG	C50H92O6	96680	59984	82582	103214	62690	72396	66856	58984	67658	46018	94524	64150
70.7	834.7519	TG 15:0_16:1_18:1	GL	TAG	C52H96O6	196934	213054	217722	229494	224462	214266	226084	212678	240938	225438	263122	247334
72.0	920.8621	TG 15:0_16:1_24:0	GL	TAG	C58H110O6	21704	11838	17010	14684	8398	8184	0	14350	8288	8824	12176	10002
71.8	862.7831	TG 15:0_18:1_18:1	GL	TAG	C54H100O6	45066	36614	58398	83022	32412	43350	35782	36894	34044	24884	54834	39038
72.2	824.7675	TG 16:0_16:0_16:0	GL	TAG	C51H98O6	106140	74570	95966	114152	77700	85676	77586	80458	71408	180570	129902	77840
71.1	822.7522	TG 16:0_16:0_16:1	GL	TAG	C51H96O6	175728	137508	173386	229546	146004	149642	151448	137258	141530	111704	213070	149636
73.5	852.7992	TG 16:0_16:0_18:0	GL	TAG	C53H102O6	88456	49356	58306	63536	46158	49544	52854	53890	46722	50510	76434	52930
72.3	850.7831	TG 16:0_16:0_18:1	GL	TAG	C53H100O6	147176	91750	123568	152036	59926	67440	63238	95820	58788	69692	104330	75446
71.2	848.7684	TG 16:0_16:0_18:2	GL	TAG	C53H98O6	246368	134694	186576	208576	114728	135954	128436	166372	120750	97582	183970	122588
69.9	820.7372	TG 16:0_16:1_16:1	GL	TAG	C51H94O6	184550	130278	162404	207646	132476	151674	148134	138410	145782	104710	215476	145416
76.5	948.8928	TG 16:0_16:1_25:0	GL	TAG	C60H114O6	11928	11286	17320	19164	12340	12316	11876	11644	11566	10338	15938	10842
74.6	880.8307	TG 16:0_18:0_18:0	GL	TAG	C55H106O6	144226	52962	56478	44726	52408	51808	51604	63904	51632	46362	84646	52204
73.5	878.8142	TG 16:0_18:0_18:1	GL	TAG	C55H104O6	48008	39258	90260	89274	33022	41554	40282	41812	35146	32774	47984	40490
71.2	874.7839	TG 16:0_18:1_18:2	GL	TAG	C55H100O6	752182	394326	291908	245322	71806	100928	92104	476556	77686	96188	111150	113346
77.3	962.909	TG 16:0_18:1_24:0	GL	TAG	C61H116O6	9936	9842	13794	17324	9078	11256	9254	8914	8956	7340	13356	9668
70.1	872.7678	TG 16:0_18:2_18:2	GL	TAG	C55H98O6	408746	163486	108776	84076	31464	44232	43398	207060	41058	41744	45144	44378
68.6	818.7208	TG 16:1_16:1_16:1	GL	TAG	C51H92O6	78770	45614	57352	75012	47274	54180	50924	45688	46546	32786	78948	54446
70.2	846.7514	TG 16:1_16:1_18:1	GL	TAG	C53H96O6	0	0	82510	103218	57310	71046	64670	0	61086	46972	96172	65866
69.2	844.7379	TG 16:1_16:1_18:2	GL	TAG	C53H94O6	23206	13516	16792	22270	13320	13754	14442	13794	12618	10438	21802	14314
75.0	894.8456	TG 17:0_18:0_18:0	GL	TAG	C56H108O6	20310	11746	14532	13460	11488	13192	11330	13016	11126	10372	14238	10876
73.6	904.8308	TG 18:0_18:1_18:1	GL	TAG	C57H106O6	45596	46566	129844	139154	32320	44060	30104	34490	32670	32492	43052	31070
75.9	934.8781	TG 18:0_18:1_20:0	GL	TAG	C59H112O6	17224	17508	39496	44040	17594	19282	17252	16966	15860	13570	25944	19754
72.3	902.8148	TG 18:1_18:1_18:1	GL	TAG	C57H104O6	208158	174198	290004	433930	73278	149270	88038	104988	74820	83832	151308	95690
71.3	900.7997	TG 18:1_18:1_18:2	GL	TAG	C57H102O6	213070	115292	124386	129764	40970	61910	56482	122288	50028	45990	63466	55062
73.5	930.8471	TG 18:1_18:1_20:1	GL	TAG	C59H108O6	15442	18224	21480	41342	7838	12610	7966	6870	5746	7428	12562	7074
71.7	926.8137	TG 18:1_18:1_20:3	GL	TAG	C59H104O6	16112	12426	14264	20994	4038	10466	6282	11442	4860	5404	6974	5072
69.0	896.7684	TG 18:2_18:2_18:2	GL	TAG	C57H98O6	73842	30258	29402	24728	15768	21330	21082	32326	19112	13792	21050	17992
68.3	880.7155	Ubidecarenone	Prenol	Quin	C59H90O4	18360	11272	14540	22614	4420	6774	7784	8636	8456	4976	7366	3804
69.2	509.5263	WE 34:0	Sterol	WE	C34H68O2	26968	16388	18024	27786	14974	19010	17832	15906	15062	11120	18826	15040
67.2	507.5117	WE 34:1	Sterol	WE	C34H66O2	56122	33488	54558	73022	31406	43612	40152	34968	32486	28782	49426	34362
66.7	505.4963	WE 34:2	Sterol	WE	C34H64O2	93112	96598	109654	166564	25036	51084	38302	16784	16692	33966	65938	28374
71.2	537.5593	WE 36:0	Sterol	WE	C36H72O2	12118	10570	11534	13920	12002	10948	10522	11164	10220	7680	11892	10338
71.3	551.5744	WE 37:0	Sterol	WE	C37H74O2	20090	19234	25630	47552	7518	19036	11618	9734	6894	11380	21466	18392
69.8	549.5589	WE 37:1	Sterol	WE	C37H72O2	43296	33480	44360	69530	0	27002	23868	0	0	0	35588	25124
68.3	547.543	WE 37:2	Sterol	WE	C37H70O2	22340	13698	22400	34386	10326	15762	13138	11322	11846	10702	16622	13104
70.8	563.574	WE 38:1	Sterol	WE	C38H74O2	65802	69334	93540	150908	19484	42082	29236	27786	0	29658	41442	31414
69.4	561.5588	WE 38:2	Sterol	WE	C38H72O2	63442	42372	79982	130382	29470	50824	42702	35516	39580	31160	60988	41168
67.7	559.543	WE 38:3	Sterol	WE	C38H70O2	13406	7640	16756	26060	6242	9616	0	6074	6852	8000	11206	9010
71.6	577.5901	WE 39:1	Sterol	WE	C39H76O2	112256	112606	161100	288960	32050	71486	63200	37190	27032	54438	113864	82414
70.3	592.6027	WE 39:2	Sterol	WE	C39H74O2	32244	22672	35372	55004	10872	19086	17222	12480	10780	13140	25398	18944
69.7	587.5745	WE 40.3	Sterol	WE	C40H74O2	17926	14124	20536	35764	6816	11614	9780	8046	6754	9066	13252	10422
73.6	593.6213	WE 40:0	Sterol	WE	C40H80O2	56288	63754	74866	129204	19302	45836	28430	31050	16098	34652	73386	54110
71.1	606.6167	WE 40:2	Sterol	WE	C40H76O2	199894	152362	190154	296986	49500	84158	92544	62624	35836	68646	145722	86880
74.3	624.6637	WE 41:0	Sterol	WE	C41H82O2	84452	81814	144106	187164	30594	73368	36518	42986	25538	48578	91430	73488
74.9	638.679	WE 42:0	Sterol	WE	C42H84O2	151006	174164	232796	394168	55704	173732	71174	85324	52532	94234	215798	146350
72.6	634.6477	WE 42:2	Sterol	WE	C42H80O2	565318	528792	702572	1158704	151102	314638	272930	188948	94934	223522	514774	272632
75.6	652.6946	WE 43:0	Sterol	WE	C43H86O2	137386	147300	245578	329812	58064	205438	64162	83476	55762	92238	202910	154776
74.5	650.6792	WE 43:1	Sterol	WE	C43H84O2	1029970	1252808	1665658	2560916	397630	925674	470886	455492	320034	647660	1133578	824086
73.5	648.6633	WE 43:2	Sterol	WE	C43H82O2	187164	191718	303060	525172	56082	139866	84174	71628	46896	98500	192994	122202
72.5	646.6479	WE 43:3	Sterol	WE	C43H80O2	21384	26086	52602	97584	8188	18158	0	0	7590	12718	25512	16540
76.3	666.7099	WE 44:0	Sterol	WE	C44H88O2	59496	62146	105456	149396	22548	86448	25228	31774	22820	36390	84992	64398
75.2	664.6946	WE 44:1	Sterol	WE	C44H86O2	1344060	1577448	2185174	3338880	584592	1623828	619780	631724	494030	870070	1596992	1059136
74.2	662.6791	WE 44:2	Sterol	WE	C44H84O2	289970	329372	462794	765334	93112	235756	120936	139886	67858	140150	229854	166184
73.1	643.6366	WE 44:3	Sterol	WE	C44H82O2	26878	30298	49798	102910	9632	22574	14060	11176	7276	15076	28958	17152
71.9	658.6481	WE 44:4	Sterol	WE	C44H80O2	24566	25194	38742	77366	8680	17702	10308	8550	6574	11564	22716	12488
75.4	690.7103	WE 46:2	Sterol	WE	C46H88O2	685256	751648	969734	1559646	244326	560414	309738	279154	177332	351576	684410	412308
74.4	688.6946	WE 46:3	Sterol	WE	C46H86O2	110712	98206	180690	288968	39162	88574	51622	47482	33426	57416	107978	70524

77.3	706.7415	WE 47:1	Sterol	WE	C47H92O2	125918	139720	196350	329868	40216	111312	42298	48536	34844	63348	132350	101132
76.1	704.7253	WE 47:2	Sterol	WE	C47H90O2	72358	71702	121406	233072	21774	55964	27108	24974	14180	33130	56004	42726
78.1	720.7571	WE 48:1	Sterol	WE	C48H94O2	54774	62272	111470	132860	19618	46704	18586	19886	14430	31902	61356	44400
76.8	718.7414	WE 48:2	Sterol	WE	C48H92O2	704790	814952	1057226	1717608	255170	521882	265856	250222	172714	372516	686860	443738
75.7	699.6984	WE 48:3	Sterol	WE	C48H90O2	22482	27826	45760	90032	8574	18434	11870	10772	6714	15760	25894	16336
77.5	715.7291	WE 49:2	Sterol	WE	C49H94O2	13018	13436	21630	46410	4272	8670	4620	3880	3410	7674	9294	7314
78.3	746.7726	WE 50:2	Sterol	WE	C50H96O2	274388	366776	555312	978952	109188	204508	96090	83672	66358	159264	282736	179626
77.0	744.7566	WE 50:3	Sterol	WE	C50H94O2	74018	85956	158028	291236	25584	61230	26188	24954	19194	41516	79094	51500
CE: cholesterol esters; WE: wax esters; GP: glycerophospholipids; GL: glycerolipid; TAG: triacylglycerol; DAG: diacylglycerol; SL: sphingolipid; SM: sphingomyelin; PK: polyketide; PC: Phosphocholine; PE: Phosphoethanolamine; PS: Phosphoinositol; SEP: Phosphoserine; Cer: Ceramide; Flav: flavonoid; Quin: quinone																	
